# A critical role of DDRGK1 in endoplasmic reticulum homoeostasis via regulation of IRE1α stability

**DOI:** 10.1038/ncomms14186

**Published:** 2017-01-27

**Authors:** Jiang Liu, Ying Wang, Lizhi Song, Linghua Zeng, Weiwei Yi, Ting Liu, Huanzhen Chen, Miao Wang, Zhenyu Ju, Yu-Sheng Cong

**Affiliations:** 1 Institute of Aging Research, School of Medicine, Hangzhou Normal University, Hangzhou, Zhejiang 310036, China; 2 Department of Cardiology, The First Hospital of Shanxi Medical University, Taiyuan, Shanxi 030001, China

## Abstract

Disturbance of endoplasmic reticulum (ER) homoeostasis induces ER stress and leads to activation of the unfolded protein response (UPR), which is an adaptive reaction that promotes cell survival or triggers apoptosis, when homoeostasis is not restored. DDRGK1 is an ER membrane protein and a critical component of the ubiquitin-fold modifier 1 (Ufm1) system. However, the functions and mechanisms of DDRGK1 in ER homoeostasis are largely unknown. Here, we show that depletion of DDRGK1 induces ER stress and enhances ER stress-induced apoptosis in both cancer cells and hematopoietic stem cells (HSCs). Depletion of DDRGK1 represses IRE1α-XBP1 signalling and activates the PERK-eIF2α-CHOP apoptotic pathway by targeting the ER-stress sensor IRE1α. We further demonstrate that DDRGK1 regulates IRE1α protein stability via its interaction with the kinase domain of IRE1α, which is dependent on its ufmylation modification. Altogether, our results provide evidence that DDRGK1 is essential for ER homoeostasis regulation.

## Abstract (web summary)

DDRGK1 is an ER membrane protein that is subject to Ufm1 modification, but its function in ER homeostasis is unknown. Here, the authors show that ufmylated DDRGK1 interacts with and stabilizes the ER-stress sensor protein IRE1a, in turn repressing ER stress and apoptosis.

The endoplasmic reticulum (ER) is an essential organelle for multiple cellular functions, including the maintenance of calcium homoeostasis; the biosynthesis of proteins, lipids or sterols; and the transport of synthesized proteins^1^,^2^. Disturbances in redox homoeostasis, calcium regulation, glucose deprivation or viral infection can lead to ER stress and trigger the unfolded protein response (UPR) to alter transcriptional and translational programs in the stressed cell^1^,^3^,^4^. In eukaryotic cells, the UPR is an adaptive cellular response to the disturbance of normal ER functions that attenuates the aggregation of unfolded or misfolded proteins and promotes cell survival. However, during prolonged or overwhelming ER stress, the UPR fails to restore ER homoeostasis, and the apoptotic cascade is activated^5^,^6^. There are three branches of the UPR that are initiated by distinct ER stress sensors located on the ER membrane: inositol-requiring enzyme 1 (IRE1α), PKR-like endoplasmic reticulum kinase (PERK) and activating transcription factor 6 (ATF6)^1^,^7^. IRE1α has been considered a master regulator of cell fate determination under ER stress. In the early stage of the UPR, IRE1α splices the mRNA of the transcription factor X-box-binding protein 1 (*XBP1*) to induce the transcription of ER quality control components to restore ER homoeostasis and promote cell survival^7^,^8^. If the restoration of ER homoeostasis fails, IRE1α represses the adaptive responses and activates apoptosis through JNK signalling^9^,^10^. Similar to IRE1α, PERK directly phosphorylates the subunit of eukaryotic initiation factor 2 (eIF2α), which attenuates global mRNA translation to protect cells from ER stress-mediated apoptosis at the initial phase of the UPR (refs 3, 11). In addition, eIF2α selectively translates activating transcription factor 4 (ATF4), which subsequently regulates several UPR target genes, including those involved in ER stress-mediated apoptosis, such as C/EBP homologous protein (*CHOP*)^5^,^9^,^11^. The ability to respond to perturbations in ER stress is a fundamental property of cells.

DDRGK domain-containing protein 1 (DDRGK1) is a newly identified ER-associated protein that is induced by ER stress^12^. The DDRGK1 protein contains a signal peptide for ER anchoring, a transmembrane helix and a proteasome component (PCI) domain. The PCI domain is a known protein-protein interaction mediator involved in several multi-protein complexes that regulates the protein life span^13^. Several studies indicate that DDRGK1 forms a large protein complex with UFL1/NLBP (Ufm1-specific ligase 1), the putative tumour suppressor C53/LZAP and Ufm1 (ubiquitin-fold modifier 1) and each of these proteins is involved in ufmylation^12^,^14^,^15^,^16^. Ufmylation is a ubiquitin-like modification, and components of the ufmylation system are induced under ER stress^12^,^17^,^18^,^19^. A recent study demonstrated that DDRGK1 is a critical component of the Ufm1 system, which targets ASC1 for oestrogen receptor (ERα) transactivation and breast cancer progression^20^. However, the biological functions of DDRGK1 are largely unknown.

In the present study, we provide evidence that DDRGK1 plays an essential role in the maintenance of ER homoeostasis. Our data show that DDRGK1 regulates IRE1α protein stability through an ufmylation-dependent protein-protein interaction. These results underscore the physiological significance of DDRGK1 and its ufmylation modification in ER homoeostasis.

## Results

### DDRGK1 is required for ER homoeostasis

**Figure 1:**
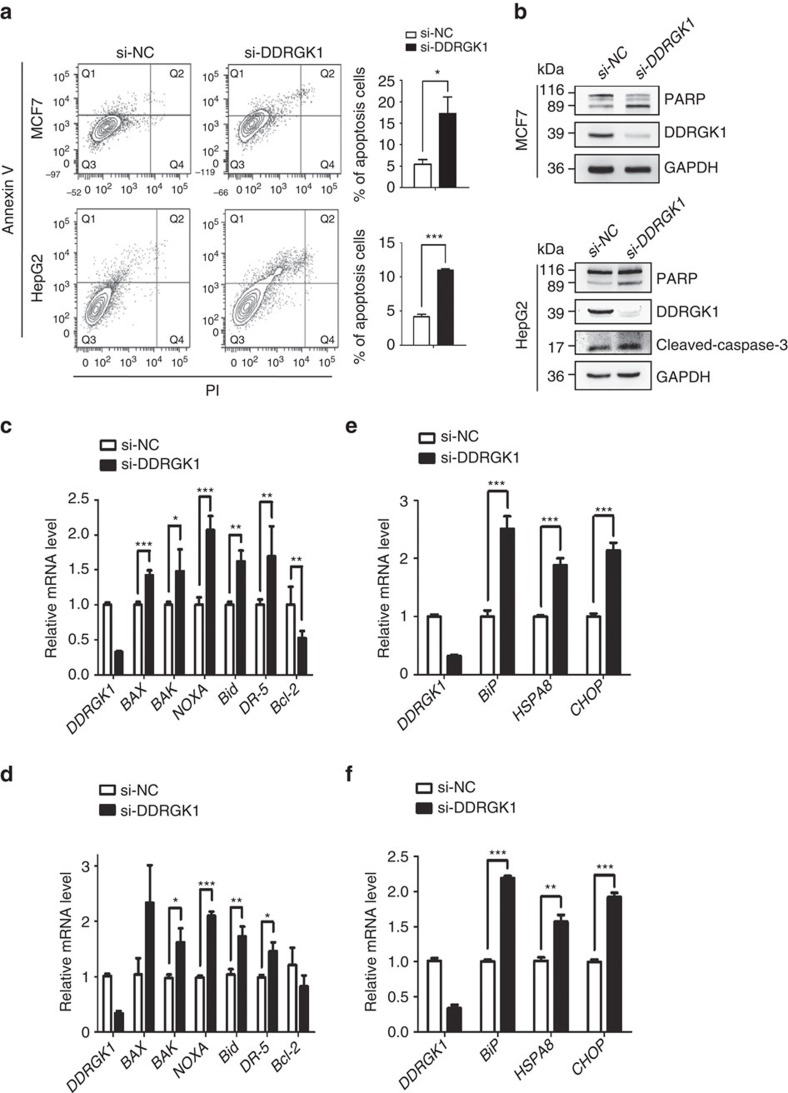
Depletion of DDRGK1 leads to apoptosis and elevated ER stress. (**a**) MCF7 and HepG2 cells were transfected with either control siRNA or siRNA targeting DDRGK1 for 72 h. The cells were subsequently stained with Annexin V and PI and subjected to flow cytometric analysis followed by the quantification of apoptotic cells (Annexin V^+^). (**b**) Western blot analysis of PARP and cleaved caspase-3 in control and DDRGK1-knockdown MCF7 and HepG2 cells described in **a**. (**c**,**d**) Q-PCR analysis of the relative mRNA expression levels of *BAX*, *BAK*, *NOXA*, *Bid*, *DR-5* and *Bcl-2* in control and DDRGK1-knockdown MCF7 and HepG2 cells. (**e**,**f**) Q-PCR analysis of the relative mRNA expression levels of *BiP*, *HSPA8* and *CHOP* in control and DDRGK1-knockdown MCF7 and HepG2 cells. All data are presented as mean±s.d. from three experiments. *P<0.05, **P<0.01 and ***P<0.001 by Student's *t*-test.

To understand the cellular function of DDRGK1, we examined the effects of DDRGK1 knockdown using a mixture of two siRNAs as described previously^21^. We found that knockdown of DDRGK1 induced apoptotic cell death in both MCF7 and HepG2 cells, characterized by Annexin V/PI staining (Fig. 1a), caspase-3 activation and PARP cleavage (Fig. 1b). It should be noted that we can detect the capspase-3 cleavage in HepG2 cells induced by knockdown of DDRGK1 but not in MCF7 cells, because MCF7 cells are caspase-3 deficient^22^. Quantitative real-time PCR (Q-PCR) analysis showed that knockdown of DDRGK1 increased the expression of the pro-apoptotic genes *BAX*, *BAK*, *NOXA*, *DR5* and *Bid*, whereas the expression of the anti-apoptotic gene *Bcl-2* was decreased (Fig. 1c,d). Importantly, we found that knockdown of DDRGK1 in MCF7 and HepG2 cells induced ER stress responses, with increased ER stress-specific gene expression of *BiP*, *HSPA8* and *CHOP* (Fig. 1e,f).

**Figure 2:**
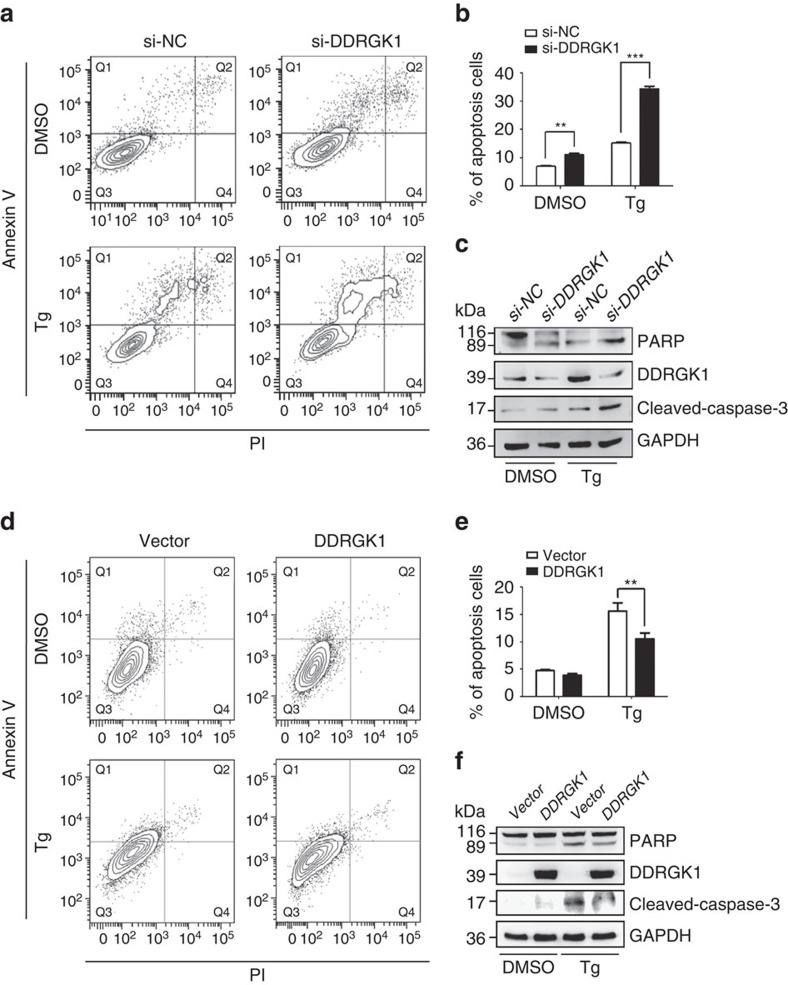
DDRGK1 plays a protective role in ER stress-induced apoptosis. (**a**). HepG2 cells were transfected with control siRNA or with siRNA against DDRGK1 for 72 h, and then the cells were treated with DMSO (vehicle control) or Tg (2.5 μM) for 24 h before harvesting. The cells were stained with Annexin V and PI, followed by flow cytometric analysis. (**b**). Quantification of the apoptotic cells (Annexin V^+^) in **a**. (**c**) Western blot analysis of PARP and cleaved caspase-3 in the HepG2 cells described in **a**. (**d**) HepG2 cells were transfected with control vector or DDRGK1 for 36 h, and the cells were treated with DMSO or Tg (2.5 μM) for 24 h before harvesting. The cells were stained with Annexin V and PI, followed by flow cytometric analysis. (**e**) Quantification of the apoptotic cells (Annexin V^+^) in **d**. (**f**) Western blot analysis of PARP and cleaved caspase-3 in the HepG2 cells described in **d**. All data are presented as mean±s.d. from three experiments. **P<0.01 and ***P<0.001 by Student's *t*-test.

To further confirm that DDRGK1 is involved in the ER stress response, we determined the effect of DDRGK1 on cell survival after treatment with the ER stress inducers thapsigargin (Tg) and tunicamycin (Tm). Knocking down DDRGK1 enhanced ER stress-induced apoptosis by Tg treatment in both HepG2 and MCF7 cells, as assessed by Annexin V/PI staining and cleavage of caspase-3 and PARP (Fig. 2a–c and Supplementary Fig. 1A–C). In contrast, over-expression of DDRGK1 reduced ER stress-induced cell death in HepG2 cells (Fig. 2d–f). In addition, the viability of MCF7 cells was assessed by MTT assay, and the results showed that knockdown of DDRGK1 rendered the cells more sensitive to Tg or Tm treatment, with reduced cell viability (Supplementary Fig. 1D), whereas over-expression of DDRGK1 promoted cell survival after Tg or Tm treatment (Supplementary Fig. 1E). Taken together, our results suggest that DDRGK1 plays a vital role in regulating ER homoeostasis.

### DDRGK1 modulates the UPR

**Figure 3:**
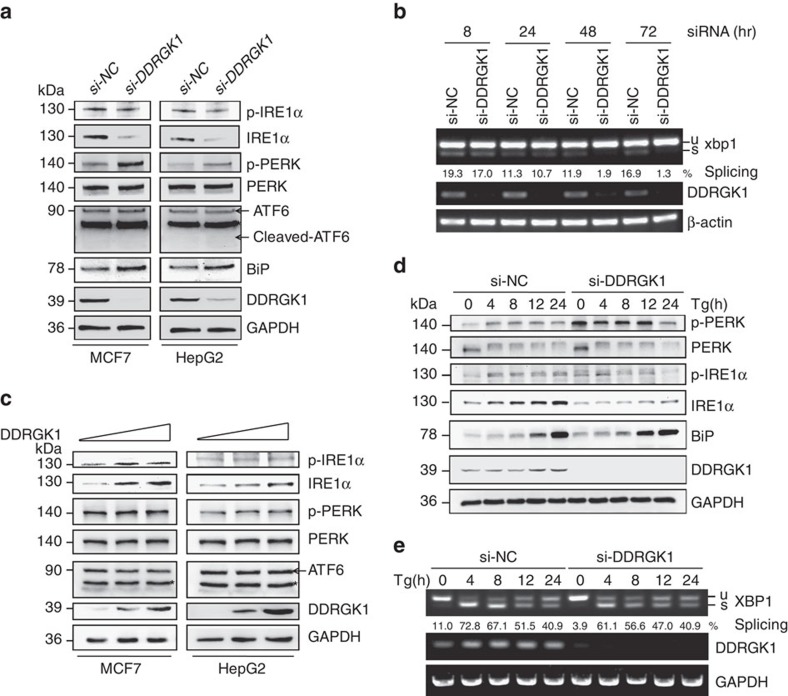
DDRGK1 modulates the UPR. (**a**) MCF7 and HepG2 cells were transfected with either control siRNA or siRNA targeting DDRGK1 for 72 h. The protein levels of p-IRE1α, IRE1α, p-PERK, PERK, ATF6 and BiP were determined by western blot. (**b**) MCF7 cells were transfected with control siRNA or siRNA against DDRGK1, and the cells were harvested at the indicated times for RT-PCR analysis of *XBP-1* splicing. (**c**) MCF7 and HepG2 cells were transfected with control or DDRGK1 vectors at various doses for 36 h. The protein levels of p-IRE1α, IRE1α, p-PERK, PERK and ATF6 were determined by western blot. (**d**) MCF7 cells were transfected with siRNA control or siRNA targeting DDRGK1 for 72 h. The cells were collected for western blot after treatment with 2.5 μM Tg at the indicated times. (**e**) RT-PCR analysis of *XBP-1* splicing in **d**. *Represents non-specific band.

To understand how DDRGK1 regulates ER homoeostasis, we first analysed changes in the protein levels of three UPR sensors and their downstream targets in DDRGK1-depleted cells. The results showed that knockdown of DDRGK1 in MCF7 and HepG2 cells significantly reduced the levels of total IRE1α but weakly decreased phosphorylated IRE1α (p-IRE1α), which may be because of the reduced levels of total IRE1α (Fig. 3a). The levels of ATF6 and cleaved ATF6 were not affected (Fig. 3a). Interestingly, knockdown of DDRGK1 did not affect the levels of total PERK, but the levels of phosphorylated PERK (p-PERK) and BiP were significantly increased (Fig. 3a). We then examined the effects of DDRGK1 depletion on the IRE1α substrate *XBP1*. The results showed that *XBP1s*, a spliced form of *XBP1*, was significantly decreased in DDRGK1-knockdown MCF7 cells in a time-dependent manner (Fig. 3b), which was correlated with changes in the IRE1α protein levels (Supplementary Fig. 2A). In addition, depletion of DDRGK1 increased the levels of eIF2α phosphorylation (p-eIF2α) and CHOP expression in both MCF7 and HepG2 cells (Supplementary Fig. 2B). These results indicate that depletion of DDRGK1 represses UPR-IRE1α signalling and activates the UPR-PERK apoptotic pathway, which consequently triggers apoptosis, as observed in Figs 1 and 2. However, the level of IRE1α was increased in DDRGK1 over-expressing cells in a dose-dependent manner (Fig. 3c), while the levels of p-PERK, PERK, ATF6, p-IRE1α, BiP and the spliced form of *XBP1* were not significantly changed (Fig. 3c; Supplementary Fig. 2C and D). To explore the functional relationship between DDRGK1 and the UPR, we assessed the dynamic changes of IRE1α and PERK in DDRGK1-knockdown MCF7 cells and in control cells following Tg treatment at different time points. Treatment with Tg increased the levels of IRE1α and p-IRE1α, p-PERK and BiP in control cells, as expected. However, the total IRE1α levels were significantly decreased (0–24 h), while the p-IRE1α levels were modestly decreased (4–24 h) in DDRGK1-depleted cells compared with control cells (Fig. 3d). Concomitantly the level of *XBP1s* was decreased in DDRGK1-depleted cells compared with control cells (4–12 h) (Fig. 3e). The levels of total PERK were not changed, but p-PERK was significantly increased in DDRGK1-depleted cells (0–12 h) (Fig. 3d). Similar results were obtained in HepG2 cells (Supplementary Fig. 2E and F). Taken together, our results suggest that depletion of DDRGK1 causes IRE1α degradation and PERK activation.

### DDRGK1 regulates the UPR by targeting IRE1α

**Figure 4:**
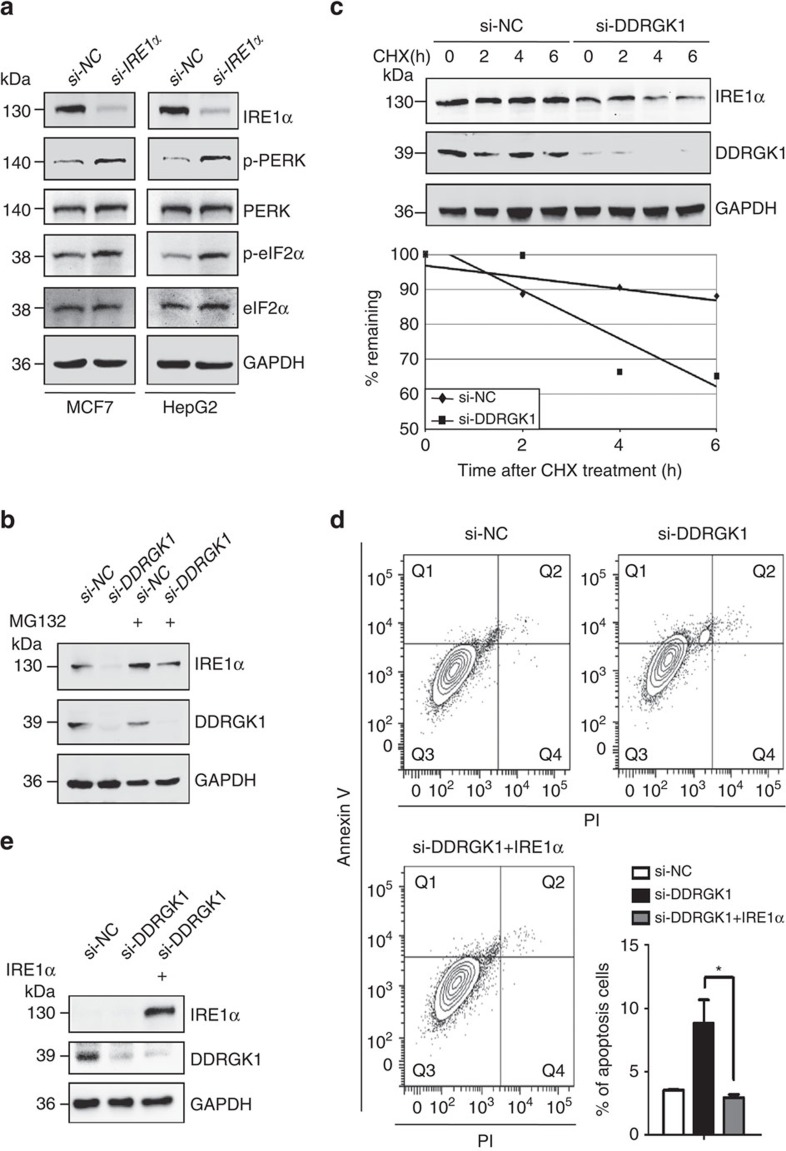
DDRGK1 regulates the UPR by targeting IRE1α. (**a**). MCF7 and HepG2 cells were transfected with either control siRNA or siRNA targeting IRE1α for 72 h. The protein levels of p-PERK, PERK, p-eIF2α and eIF2α were determined by western blot. (**b**). Western blot analysis of IRE1α in control and DDRGK1-knockdown MCF7 cells treated with or without MG132 (20 μM, 8 h). (**c**). Western blot analysis of IRE1α decay in control and DDRGK1-knockdown MCF7 cells after treatment with 100 μg ml^−1^ cycloheximide for the indicated times. The graph represents the quantification of the IRE1α protein levels. (**d**) MCF7 cells were transfected with either control siRNA or siRNA targeting DDRGK1 for 72 h. Before harvesting, the DDRGK1-knockdown cells were transfected with either control or IRE1α vectors for 36 h. The cells were subsequently stained with Annexin V and PI and subjected to flow cytometric analysis, followed by quantification of apoptotic cells (Annexin V^+^). All data are presented as mean±s.d. from three experiments. *P<0.05 by Student's *t*-test. (**e**) Western blot analysis of IRE1α in MCF7 cells in **d**.

The UPR is triggered by ER stress sensors upon ER stress to regulate ER homoeostasis^8^. It has been reported that hepatocyte-specific deletion of IRE1α in mice resulted in activation of the UPR-PERK pathway^23^. To address whether the induction of p-PERK observed in DDRGK1-depleted cells was mediated by decreased levels of IRE1α, we examined the levels of p-PERK, PERK and its downstream targets in IRE1α-depleted cells. The results showed that knockdown of IRE1α significantly increased the protein levels of p-PERK and p-eIF2α in both MCF7 and HepG2 cells (Fig. 4a), similar to those observed in DDRGK1-depleted cells (Fig. 3a; Supplementary Fig. 2B). These results indicate that DDRGK1 regulates the UPR by targeting IRE1α. We then investigated how DDRGK1 regulates IRE1α. Our results showed that IRE1α protein levels, but not mRNA levels (Supplementary Fig. 3A), were dramatically decreased in DDRGK1-knockdown cells (Fig. 3a). Treatment of the cells with the proteasome inhibitor MG132 blocked the DDRGK1-mediated reduction of IRE1α protein (Fig. 4b; Supplementary Fig. 3B). Furthermore, we observed that depletion of DDRGK1 dramatically reduced the half-life of IRE1α in a cycloheximide chase assay (Fig. 4c). In accordance with these observations, we found that ectopic expression of IRE1α improved cell survival in both DDRGK1-depleted MCF7 and HepG2 cells compared with control cells, characterized by Annexin V/PI staining and caspase-3 activation (Fig. 4d,e; Supplementary Fig. 3C and D). Altogether, these results suggest that DDRGK1 regulates the stability of IRE1α and thereby regulates the UPR.

### DDRGK1 interacts with IRE1α

**Figure 5:**
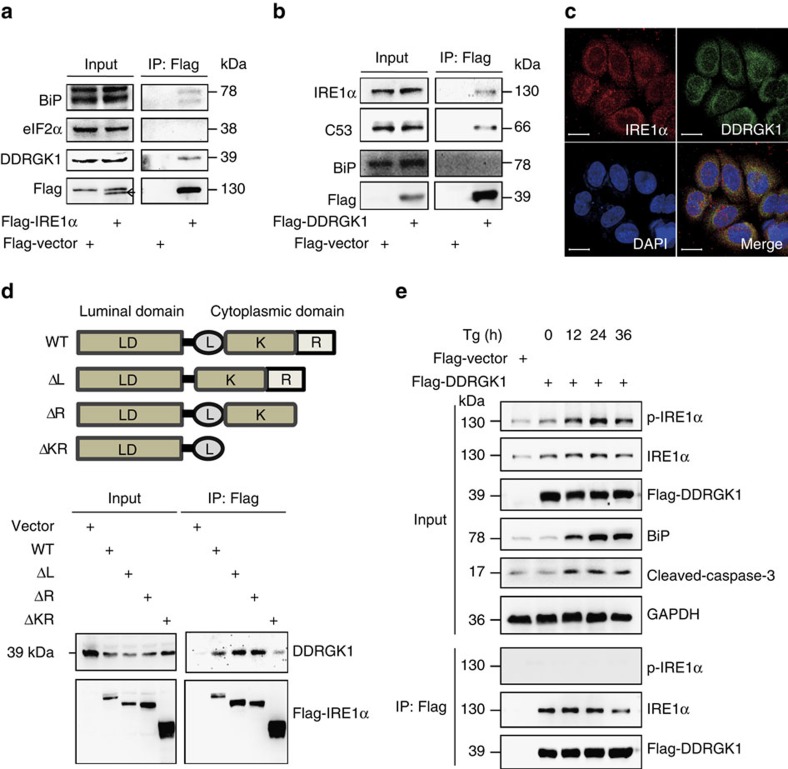
DDRGK1 interacts with IRE1α. (**a**). Western blot analysis of immunoprecipitates of Flag M2 affinity gel in HEK293T cells transfected with Flag-Vector or Flag-IRE1α vectors for 36 h. (**b**). Western blot analysis of immunoprecipitates of Flag M2 affinity gel in HEK293T cells transfected with Flag-Vector or Flag-DDRGK1 for 36 h. (**c**). MCF7 cells were double immunostained with anti-DDRGK1 antibody (green) and anti-IRE1α antibody (red). The cell nuclei were counterstained with DAPI (blue). The co-localization between the two endogenous proteins DDRGK1 and IRE1α is shown in the merge panel. Scale bar, 20 μm. (**d**) Schematics of IRE1α wild-type and truncated constructs and western blot analysis of the Flag M2 affinity gel immunoprecipitates in HEK293T cells transfected with Flag-Vector or Flag-IRE1α wild-type and truncates. (**e**) Western blot analysis of Flag M2 affinity gel immunoprecipitates in mock-or Tg-treated (2.5 μM) HEK293T cells expressing Flag-Vector or Flag-DDRGK1.

To understand how DDRGK1 regulates IRE1α stability, we tested whether DDRGK1 interacts with IRE1α via a reciprocal immunoprecipitation (IP) assay in HEK293T cells. The results showed that exogenously expressed Flag-IRE1α specifically interacted with endogenous DDRGK1 and BiP (Fig. 5a). Consistently, exogenously expressed Flag-DDRGK1 specifically interacted with endogenous IRE1α and the known DDRGK1-associated protein C53 (ref. 14). However, we were unable to detect BiP in the Flag-DDRGK1 immunoprecipitates (Fig. 5b), which suggests that DDRGK1 may not directly interact with BiP. IRE1α, as an endogenous ER-associated degradation (ERAD) substrate, is degraded through the association between IRE1α and the Sel1L-Hrd1 ERAD complex in a BiP-dependent manner^24^. Therefore, we examined whether BiP is involved in the interaction between DDRGK1 and IRE1α. The results showed that the interaction between DDRGK1 and IRE1α was not affected by knockdown of BiP (Supplementary Fig. 4). To further confirm the interaction of DDRGK1 with IRE1α, we performed immunofluorescence staining experiments. The results showed that these two proteins co-localized in the ER (Fig. 5c). To map the region involved in the interaction of IRE1α with DDRGK1, we co-transfected Flag-IRE1α wild-type and truncation mutants along with DDRGK1 into HEK293T cells, and the IP results showed that the cytoplasmic kinase domain of IRE1α was necessary for its interaction with DDRGK1 (Fig. 5d). To understand the dynamic changes in the interaction between DDRGK1 and IRE1α under ER stress conditions, HEK293T cells were transfected with Flag-DDRGK1 and treated with Tg for different time points. As expected, we observed that total IRE1α, p-IRE1α and BiP were significantly increased under ER stress. Interestingly, we found that DDRGK1 specifically interacted with unphosphorylated IRE1α, but not with p-IRE1α, in the immunoprecipitates (Fig. 5e). These results suggest that DDRGK1 interacts with and maintains the stability of non-phosphorylated IRE1α.

### Ufm1 is required for the interaction of DDRGK1 and IRE1α

**Figure 6:**
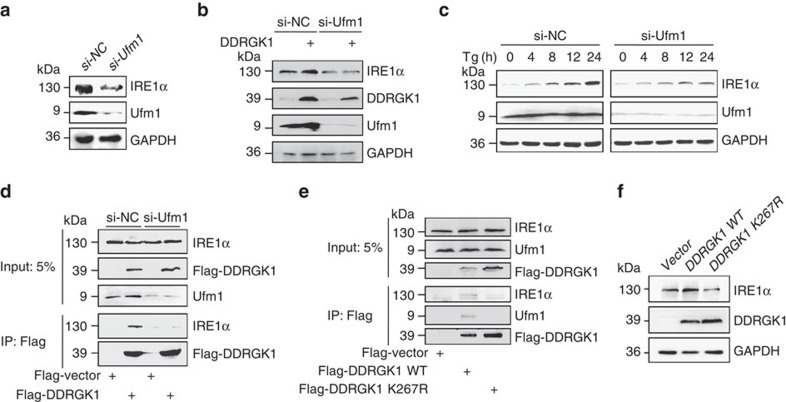
Ufmylation is required for the interaction between DDRGK1 and IRE1α. (**a**) MCF7 cells were transfected with either control siRNA or siRNA targeting Ufm1 for 72 h. The protein levels of IRE1α were determined by western blot. (**b**) Western blot analysis of IRE1α in control and Ufm1-knockdown MCF7 cells expressing vector or DDRGK1. (**c**) The protein level of IRE1α was determined by western blot in control and Ufm1-knockdown MCF7 cells after treatment with 2.5 μM Tg for the indicated times. (**d**) Western blot analysis of Flag M2 affinity gel immunoprecipitates in HEK293T control and Ufm1-knockdown cells expressing Flag-Vector or Flag-DDRGK1. (**e**) Western blot analysis of Flag M2 affinity gel immunoprecipitates in HEK293T cells transfected with Flag-Vector, Flag-DDRGK1 WT or K267R mutant. (**f**) Western blot analysis of IRE1α in MCF7 cells transfected with Flag-Vector, Flag-DDRGK1 WT or K267R mutant.

A recent study demonstrated that DDRGK1 is an ufmylation substrate and is required for ASC1 ufmylation^15^,^20^. To investigate whether ufmylation is involved in the regulation of IRE1α stability by DDRGK1, we examined whether Ufm1 depletion affects IRE1α protein expression. Similar to the depletion of DDRGK1, we observed that knockdown of Ufm1 expression dramatically reduced the levels of IRE1α protein (Fig. 6a), indicating that DDRGK1 regulates IRE1α through ufmylation. Consistently, we found that over-expression of DDRGK1 failed to stabilize the IRE1α protein in Ufm1-knockdown MCF7 cells compared with control cells (Fig. 6b). In addition, IRE1α protein levels were dramatically decreased in Ufm1-knockdown MCF7 cells in both the absence and presence of Tg (Fig. 6c), which suggests that ufmylation is required for the regulation of IRE1α protein stability by DDRGK1. We then wondered whether IRE1α is subject to ufmylation modification. To address this question, we detected the ufmylation of IRE1α in cells expressing DDRGK1 and Ufm1 as well as UBA5 (E1), UFC1 (E2) and UFL1 (E3), as previously described^20^. However, we were unable to detect any ufmylation of the IRE1α protein, although ufmylation of the DDRGK1 protein was readily detectable, as previously described (data not shown)^15^,^20^,^25^, suggesting that IRE1α might not be a target of ufmylation. Interestingly, we found that the association between DDRGK1 and IRE1α was significantly decreased in Ufm1-knockdown HEK293T cells compared with control cells (Fig. 6d). Consistent with this observation, we found that the interaction of DDRGK1 K267R (a DDRGK1 mutant deficient in ufmylation, in which the lysine residue 267 was substituted with an arginine residue)^15^ with IRE1α was dramatically decreased compared with the wild-type DDRGK1 (Fig. 6e), indicating that ufmylation of DDRGK1 is required for the association between DDRGK1 and IRE1α. Furthermore, DDRGK1 K267R failed to stabilize the IRE1α protein compared with wild-type DDRGK1 (Fig. 6f). Taken together, these results suggest that ufmylation of DDRGK1 at K267 is required for its interaction with IRE1α and the regulation of IRE1α protein stability.

### DDRGK1 is required for HSC reconstitution ability in mice

**Figure 7:**
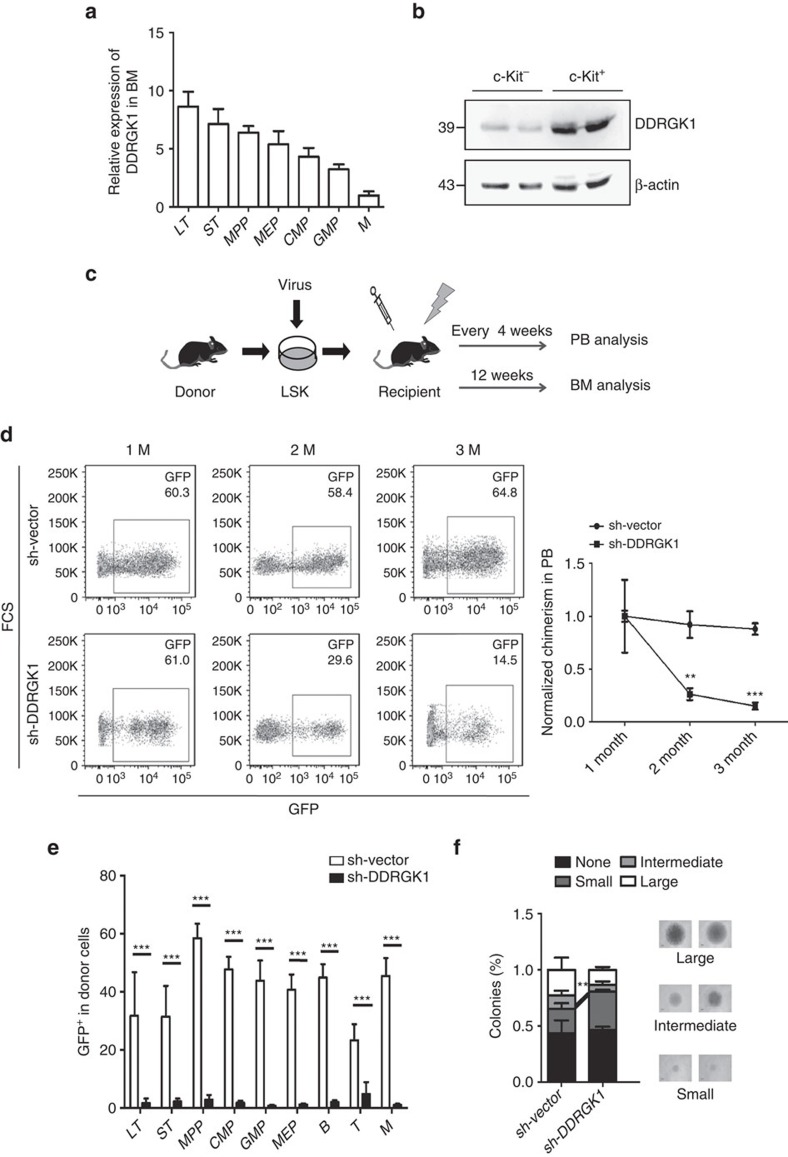
Knockdown of DDRGK1 impairs the reconstitution ability of murine HSCs. (**a**) Q-PCR analysis of the relative mRNA expression levels of *DDRGK1* in the indicated subpopulations of BM from young wild-type mice (2 months old, *n*=3). The relative expression of *DDRGK1* was normalized to GAPDH. Data are presented as mean±s.d. (**b**) Western blot analysis of DDRGK1 in enriched Lineage^+^ c-Kit^−^ and Lineage^-^ c-Kit^+^ cells from young wild-type mice (2 months old). (**c**) Experimental schematic for the reconstitution assay. (**d**) Representative FACS pattern showing the percentage of GFP-positive cells in donor-derived control (sh-Vector) or DDRGK1-knockdown (sh-DDRGK1) PB cells (Left panel). The values represent the normalized percentages of donor-derived GFP^+^ cells in the total engraftment (Right panel, *n*=3). Data are presented as mean±s.d. **P<0.01 and ***P<0.001 by Student's *t*-test. (**e**) The values represent the percentages of donor-derived GFP^+^ cells in LT-HSC (CD34^-^Flt3^-^ LSK), ST-HSC (CD34^+^Flt3^-^ LSK), MPP (CD34^+^Flt3^+^ LSK), CMP (CD34^+^CD16/32^-^Sca1^-^c-Kit^+^Lin^−^), GMP (CD34^+^CD16/32^+^Sca1^-^c-Kit^+^Lin^−^), MEP (CD34^-^ CD16/32^-^Sca1^-^c-Kit^+^Lin^−^), B, T and myeloid lineage cell populations in the BM from primary recipient mice 12 weeks after transplantation (*n*=3). Data are presented as mean±s.d. ***P<0.001 by Student's *t*-test. (**f**) Single donor-derived GFP^+^ LT-HSCs from recipient mice were sorted into 96-well plates and cultured for 14 days *in vitro*. The percentage of colonies was calculated by dividing the number of colonies by the original number of single cells that were seeded. Data are presented as mean±s.d. **P<0.01 by Student's *t*-test.

A recent study suggested that hematopoietic stem cells (HSCs) are extremely sensitive to ER stress^26^. This led us to speculate that DDRGK1 might be critical in HSC function. We first determined the expression level of *DDRGK1* across multiple hematopoietic lineages from 2-month-old wild-type mice. Q-PCR revealed a significantly higher level of *DDRGK1* expression in HSCs, including long-term HSCs (LT-HSCs), short-term HSCs (ST-HSCs) and multipotent progenitors (MPPs) (Fig. 7a). In addition, the protein level of DDRGK1 was highly expressed in HSC-enriched Lineage^-^c-Kit^+^ bone marrow (BM) cells compared with Lineage^+^c-Kit^-^ BM cells (Fig. 7b). These observations suggest an important role of DDRGK1 in stem cells. To examine the role of DDRGK1 in HSC function, competitive transplantation experiments were performed with HSCs following lentiviral knockdown of DDRGK1 (Fig. 7c). Lineage^-^Sca1^+^c-Kit^+^ (LSK) cells from CD45.1 donor mice were transduced with the control or DDRGK1-knockdown lentivirus and transplanted into lethally irradiated CD45.2 recipient mice. Flow cytometric analysis showed that the percentage of donor-derived peripheral blood (PB) cells was significantly reduced in the DDRGK1-knockdown group compared with the control, indicating that knockdown of DDRGK1 significantly impaired the competitive reconstitution ability of HSCs (Fig. 7d). While in a non-competitive transplantation experiment, the recipient mice transplanted with DDRGK1-knockdown HSCs were still alive 3 months after transplantation, this suggests that knockdown of DDRGK1 was merely compromising the competitive reconstitution capacity of HSCs. To exclude off-target effects, we designed another shRNA targeting DDRGK1 and observed similar results (Supplementary Fig. 5A). Three months after transplantation, the recipient mice were euthanized for BM analysis and *in vitro* colony-forming assays. Flow cytometric analysis showed that knockdown of DDRGK1 dramatically impaired the maintenance of transduced HSCs without affecting their lineage potential (that is, myeloid, B cell and T cell lineages), as indicated by a decreased frequency of donor-derived hematopoietic stem and progenitor cells, as well as differentiated hematopoietic cells in the BM and PB (Fig. 7e). A similar result was observed in an independent experiment using another shRNA targeting DDRGK1 (Supplementary Fig. 5B). In addition, we isolated donor-derived CD34^-^Flt3^-^LSK cells (LT-HSCs) from the recipient mice and performed a single-cell colony-forming assay. The results showed that knockdown of DDRGK1 impaired the ability of HSCs to form intermediate and large colonies (Fig. 7f). Altogether, these data suggest that DDRGK1 is required for the maintenance of HSC function.

**Figure 8:**
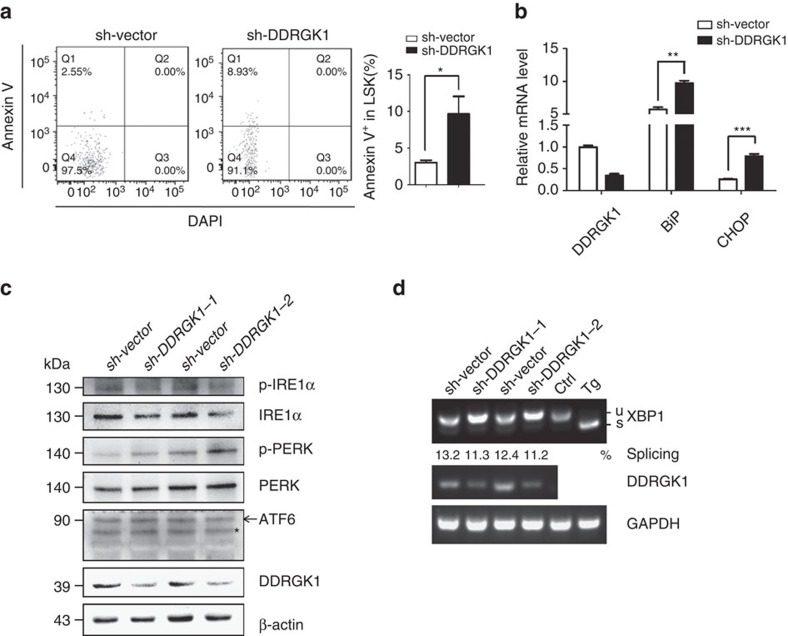
Knockdown of DDRGK1 induces ER stress in HSCs. (**a**) Representative FACS pattern showing the percentage of Annexin V-positive cells within the donor-derived control and DDRGK1-knockdown LSK cells after transplantation (Left panel). Bar graph shows the percentage of Annexin V-positive cells within LSK cells (Right panel, *n*=3). Data are presented as mean±s.d. *P<0.05 by Student's *t*-test. (**b**) Q-PCR analysis of the relative mRNA expression levels of *DDRGK1*, *BiP* and *CHOP* in donor-derived control and DDRGK1-knockdown LSK cells after transplantation (*n*=3). Data are presented as mean±s.d. **P<0.01 and ***P<0.001 by Student's *t*-test. (**c**) Western blot analysis of p-IRE1α, IRE1α, p-PERK, PERK and ATF6 in donor-derived GFP^+^Lineage^-^c-Kit^+^ cells enriched from control or DDRGK1-knockdown recipient mice. *Represents non-specific band. (**d**) RT-PCR analysis of *XBP-1* splicing in donor-derived control and DDRGK1-knockdown LSK cells after transplantation. MEF cells with Tg treatment served as an *XBP-1* splicing control.

To examine whether impaired HSC function was because of reduced homing efficiency, we labelled control or sh-DDRGK1 lentiviral transduced LSK cells purified from 2-month-old mice with fluorescent dye (violet), and injected them into lethally irradiated recipients. The percentage of labelled LSK cells present in the recipient BM 18 h after transplant was analysed by flow cytometry. The results showed that the homing ability of DDRGK-knockdown LSK cells was comparable to that of control cells (Supplementary Fig. 6A and B). To determine whether knockdown of DDRGK1 had an effect on the cell cycle status of LSK cells, we stained donor-derived control and DDRGK1-knockdown LSK cells with the proliferation marker Ki67 and DAPI and found no significant difference in the cell cycle profiles between DDRGK1-knockdown HSCs and control HSCs (Supplementary Fig. 7A). We then examined the effect of DDRGK1-knockdown on HSCs apoptosis using Annexin V staining and found a significantly higher frequency of apoptosis in donor-derived DDRGK1-knockdown LSK cells compared with control LSK cells; this phenomenon was observed with both of the shRNAs targeting DDRGK1 (Fig. 8a; Supplementary Fig. 7B). Next, we examined the levels of reactive oxygen species (ROS) in the transduced HSCs. The results showed that the ROS level was comparable between the control and DDRGK1-knockdown groups (Supplementary Fig. 7C). On the basis of above observations, we concluded that knockdown of DDRGK1 in HSCs induced apoptotic cell death, thereby impairing HSC function.

To investigate whether the impaired HSC function was because of activation of the ER stress response in DDRGK1-knockdown HSCs, we analysed the expression levels of *BiP* and *CHOP* by Q-PCR in control and DDRGK-knockdown engrafted HSCs, the results showed that the mRNA levels of *BiP* and *CHOP* were significantly increased in DDRGK1-knockdown HSCs (Fig. 8b). In addition, we examined the protein levels of ER stress sensors in the control and DDRGK-knockdown Lineage^-^c-Kit^+^ BM cells. In accordance with the observation in cancer cell lines, knockdown of DDRGK1 showed decreased protein levels of IRE1α and p-IRE1α and an increased level of p-PERK, whereas the ATF6 levels did not differ in the control and DDRGK1-knockdown groups (Fig. 8c). Consistently, the level of *XBP1*s was decreased in donor-derived DDRGK1-knockdown LSK cells (Fig. 8d). Taken together, these results indicate that knockdown of DDRGK1 impaired IRE1α signalling but activated the PERK pathway and further support that DDRGK1 is critical for the proper maintenance of ER homoeostasis and thereby essential for the survival and maintenance of HSCs.

## Discussion

**Figure 9:**
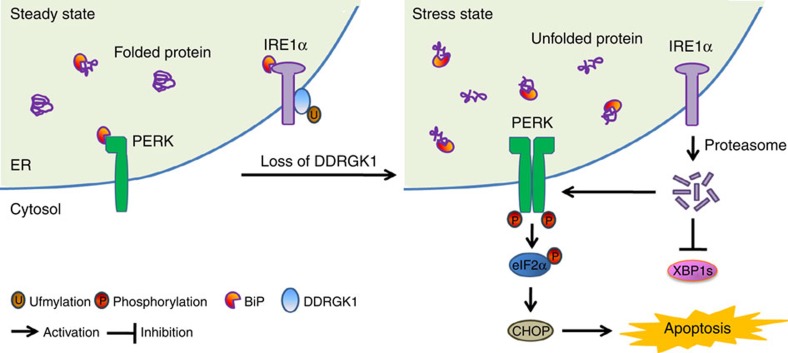
Regulatory functions of DDRGK1 in the maintenance of ER homoeostasis. IRE1α is stabilized through a physical interaction with DDRGK1 under homeostatic conditions. The loss of DDRGK1 decreases the protein abundance of IRE1α and activates the PERK-eIF2α-CHOP apoptotic pathway.

In the present study, we provide evidence that DDRGK1 is involved in regulating ER homoeostasis both *in vitro* and *in vivo*. We demonstrate that depletion of DDRGK1 induces ER stress and enhances ER stress-induced apoptosis in both cancer cells and HSCs. Our results indicate that DDRGK1 controls IRE1α protein stability via its interaction with the kinase domain of IRE1α, which is dependent on ufmylation at K267 of DDRGK1. Loss of DDRGK1 accelerates IRE1α protein degradation and promotes PERK activation, thereby inducing cell death. Thus, these findings establish the essential role of DDRGK1 and Ufm1 modification in maintaining ER homoeostasis and provide critical insight into the regulatory mechanisms of ER homoeostasis, as illuminated in Fig. 9.

Different physiological and pathological perturbations interfere with protein folding processes, leading to ER stress. There is substantial evidence for the involvement of chronic ER stress in many diseases, including diverse forms of cancer, neurodegeneration, diabetes and proinflammatory conditions^27^. The UPR is an adaptive response that facilitates protein folding, processing, export and degradation during ER stress. Although the initial phase of the UPR serves as a cytoprotective mechanism to rebalance ER homoeostasis, persistent activation of the UPR triggers apoptosis^2^,^28^,^29^. One of our important findings is that DDRGK1 regulates the steady-state levels of the key ER stress sensor IRE1α. IRE1α has long been considered a positive regulator of cell survival, and its activities are attenuated by persistent ER stress; however, PERK signalling, including translational inhibition by activation of eIF2α and induction of the proapoptotic transcription regulator *CHOP*, is maintained^7^. Interestingly, we observed that depletion of DDRGK1 resulted in the activation of PERK signalling (Fig. 3; Supplementary Fig. 2), which has similar effects on the UPR as IRE1α depletion. Furthermore, ectopic expression of IRE1α rescued DDRGK1-depleted cells from cell death (Fig. 4). These results imply that DDRGK1 depletion induces IRE1α degradation, which leads to activation of the UPR and apoptotic cell death.

Ufm1 is a recently identified protein modifier^25^, and the targets and physiological functions of the Ufm1 system are largely unknown. Our results show that ufmylation participates in the regulation of IRE1α protein stability by DDRGK1. Although ufmylation might not be responsible for IRE1α degradation, it is required for the interaction of DDRGK1 with IRE1α. Because the ufmylation of DDRGK1 on K267 is required for the association between DDRGK1 and IRE1α, substitution of a lysine residue with an arginine residue at position 267 (DDRGK1 K267R) impaired the interaction between DDRGK1 and IRE1α. The remaining question is how this interaction affects IRE1α degradation. One possibility could be that the interaction of DDRGK1 with IRE1α counteracts ubiquitylation and subsequently inhibits the ERAD-mediated degradation of IRE1α (ref. 23). It is also possible that the interaction of DDRGK1 with IRE1α might affect its endoribonuclease and kinase activity. However, the detailed mechanisms for the regulation of IRE1α protein stability by DDRGK1 and ufmylation require further investigation. Nonetheless, our results demonstrate that DDRGK1 plays a critical role in the maintenance of ER homoeostasis by modulating IRE1α protein stability.

Consistent with *in vitro* studies in cancer cell lines, our *in vivo* results indicate that DDRGK1 is critical for the survival of HSCs, as knockdown of DDRGK1 leads to elevated ER stress and apoptosis in HSCs. It has been reported that HSCs and other progenitor cells exhibit distinct cellular responses to extracellular ER stressors, and the UPR plays a critical role in governing the integrity of the HSC pool during stress^26^. Previous studies suggested that the Ufm1 conjugation system is essential for murine embryonic erythropoiesis^30^,^31^. A recent study showed that UFBP1 (DDRGK1) knockout mice exhibited defective erythroid development and somatic ablation of UFBP1 impaired adult hematopoiesis^32^, which is consistent with our observation that depletion of DDRGK1 impaired HSC function via increased ER stress and activation of the UPR. Importantly, our results from both *in vitro* and *in vivo* studies provide mechanistic evidence that DDRGK1 plays a critical role in ER homoeostasis. We demonstrate that DDRGK1 regulates IRE1α protein stability via its interaction with IRE1α, which is dependent on ufmylation. Given that DDRGK1 and the Ufm1 system are mostly associated with subcellular membranes and are subject to ufmylation, it is conceivable that DDRGK1-mediated ufmylation may constitute a novel protein network that is broadly involved in cellular homoeostasis. Further studies will elucidate the regulatory mechanisms and cellular functions of these large protein complexes and their implications in human diseases.

## Methods

### Cell culture and reagents

HEK293T, MCF7 and HepG2 cells were from ATCC and maintained in Dulbecco's modified Eagle's medium (DMEM) with 10% FBS (Biological Industries). Sorted LSK cells from BM were cultured in SFEM medium (STEMCELL Technologies) supplemented with 100 ng ml^−1^ of mSCF and hTPO. The antibodies used in this study included anti-Flag (Sigma, F7425, dilution 1:2,000) and anti-DDRGK1 (Sigma, HPA013373, dilution 1:1,000; Proteintech, 21445-1-AP, dilution 1:1,000), anti-CHOP (Abcam, ab11419, dilution 1:2,000), anti-Ufm1 (Abcam, ab109305, dilution 1:1,000), anti-PERK (Cell Signalling Technology, 3192S, dilution 1:1,000), anti-IRE1α (Cell Signaling Technology, 3294S, dilution 1:1,000), anti-eIF2α (Cell Signaling Technology, 9722S, dilution 1:1,000), anti-phospho-eIF2α (Cell Signaling Technology, 3597S, dilution 1:1,000), anti-BiP (Cell Signaling Technology, 3177S, dilution 1:1,000), anti-PARP (Cell Signaling Technology, 9542S, dilution 1:1,000), anti-cleaved caspase-3 (Cell Signaling Technology, 9661S, dilution 1:1,000), anti-ATF6 (Novus Biologicals, NBP1-40256, dilution 1:1,000), anti-phospho-IRE1α (Novus Biologicals, NB100-2323, dilution 1:1,000), anti-phospho-PERK (Santa Cruz Biotechnology, sc-32577, dilution 1:1,000), anti-C53 (Bethyl Laboratories, A300-871A, dilution 1:1,000), anti-GAPDH (HuaAn Biotechnology, M1310-2, dilution 1:5,000) and anti-β-actin (HuaAn Biotechnology, M1210-2, dilution 1:5,000). Western blotting was performed according to standard procedures. Uncropped scans of the blots are shown in Supplementary Fig. 8. The siRNAs targeting DDRGK1, IRE1α, BiP and Ufm1 were purchased from GenePharma, Inc. Thapsigargin (Tg) and tunicamycin (Tm) were purchased from Gene Operation.

### Cell line authentication

STR DNA profiling analysis was used for cell line authentication. MCF7 and HepG2 cell lines were found 100% matched with MCF7 and HepG2 cell lines in the ATCC and DSMZ databases, no cross-contamination of other human cells was found. The mycoplasma status of cells was tested and no mycoplasma contamination was found.

### Plasmid constructs and transfection

The mammalian expression vector p3 × FLAG-CMV (Sigma) was used to express Flag-tagged DDRGK1. A lysine-to-arginine mutation in DDRGK1 was generated by site-directed mutagenesis at residue Lys-267. pCMV-Tag4B-IRE1α wild-type and IRE1α deletion mutants were kindly provided by Dr Yong Liu (Key Laboratory of Nutrition and Metabolism, Institute for Nutritional Sciences, Shanghai Institutes for Biological Sciences, Chinese Academy of Sciences). Lentiviral vectors expressing specific shRNAs were constructed using the SF-LV-EGFP vector. The following shRNAs were used: mDDRGK1 shRNA-1: 5′-GGTGTTAGCGAAACCATGACT-3′; mDDRGK1 shRNA-2: 5′-GGCGGGAGCACGAGGAGTACC-3′. All of the constructs were verified by DNA sequencing. Plasmid transfection was performed with Turbofect (Fermentas), and RNA interference was performed with Lipofectamine 2,000 transfection reagent (Invitrogen), according to the manufacturer's instructions.

### Lentivirus production

Lentiviruses were produced in HEK293T cells after the transfection of lenti-shRNA constructs with pspAX2 and pMD2G. The viruses were concentrated by centrifugation at 25,000 r.p.m. for 2.5 h at 4 °C, and the virus pellet was subsequently suspended in SFEM medium.

### Q-PCR and reverse transcription PCR (RT-PCR)

Total RNA was extracted from cells using the TRIzol reagent/RNeasy Micro kit (Invitrogen) according to the manufacturer's instructions, followed by cDNA preparation using M-MLV reverse transcriptase (Promega). Q-PCR was performed in duplicate using SYBR Green Supermix (Bio-Rad). RT-PCR was performed using KOD-Plus-Neo Polymerase mix (Toyobo Life Science). The RT-PCR products were separated by electrophoresis in 3% or 1% agarose gels and were visualized using ethidium bromide. The sequences of the primers used for Q-PCR/RT-PCR are described in Supplementary Table 1.

### Single-cell colony-forming assay

Freshly isolated CD34^-^Flt3^-^LSK (LT-HSCs) cells were individually sorted into 96-well plates and cultured for 14 days in liquid medium. The colonies were counted and photographed.

### Flow cytometric analysis

BM cells were isolated by crushing the bones from femurs, tibiae and iliac crests, and were incubated in a lineage cocktail containing antibodies against CD4 (RM4-5, 1:100), CD8 (53-6.7, 1:100), Ter-119(TER-119, 1:100), CD11b (M1/70, 1:150), Gr-1 (RB6-8C5, 1:150) and B220 (RA3-6B2, 1:100) for 30 min. After washing, the cells were incubated in CD34 (RAM34, 1:100), CD45.1 (A20, 1:100), CD45.2 (104, 1:100), Flt3(A2F10, 1:100), Sca1 (E13-161.7, 1:100), c-Kit (ACK2, 1:100), CD16/32 (93, 1:100) and streptavidin. All monoclonal antibodies were from BD Biosciences or eBioscience, as previously described^33^. The Annexin V Apoptosis Detection kit (eBioscience or BD Biosciences) was used for apoptosis analyses. For the cell proliferation assay, BM cells were fixed and permeabilized with BD Cytofix/Cytoperm (BD Biosciences), after staining with Ki-67 antibody (BD Biosciences, B56, dilution 1:50) and then resuspended with PBS containing DAPI before analysing by fluorescence-activated cell sorting (FACS). For the *in vivo* homing assays, we labelled equal numbers of sh-Vector or sh-DDRGK1 lentiviral-transduced LSK cells with 5 μM CellTrace Violet Cell Proliferation reagent (Invitrogen) for flow cytometry. The cells were then transplanted into lethally irradiated recipients for flow cytometric analysis 18 h post-transplantation. The CellROX Deep Red Flow Cytometry Assay kit (Invitrogen) was used for the ROS analyses. We labelled equal numbers of donor-derived control or DDRGK1-knockdown BM cells with 500 nM CellROX Deep Red reagent for 60 min at 37 °C. Prepared samples were sorted on a BD Influx cell sorter (BD Bioscience) or analysed on a BD LSR Fortessa cell analyser (BD Biosciences).

### HSC reconstitution assay

LSK cells were purified from donor mice and transduced with sh-DDRGK1 or sh-Vector lentivirus. The reconstitution analysis was performed by transplanting the infected cells into lethally irradiated recipient mice every 4 weeks after transplantation. PB was collected from the recipient mice and analysed for chimerism and lineage distribution. The BM of the recipient mice was analysed 3 months after transplantation.

### Statistical analysis

Experiments were repeated at least three times. Statistical analyses were performed using a two-tailed, paired Student's *t*-test. *P*<0.05 was considered significant. *P<0.05, **P<0.01, and ***P<0.001.

### Data availability

The data that support the conclusions of this study are available from the corresponding author on request.

## 

## Additional information

**How to cite this article:** Liu, J. *et al*. A critical role of DDRGK1 in endoplasmic reticulum homoeostasis via regulation of IRE1α stability. *Nat. Commun.* **8,** 14186 doi: 10.1038/ncomms14186 (2017).

**Publisher's note:** Springer Nature remains neutral with regard to jurisdictional claims in published maps and institutional affiliations.

## Supplementary Material

Supplementary Information Supplementary figures and supplementary table.

## References

[b1] Brown M. K. & Naidoo N. The endoplasmic reticulum stress response in aging and age-related diseases. Front. Physiol. 3, 263 (2012). 22934019 10.3389/fphys.2012.00263 PMC3429039

[b2] Hetz C. The unfolded protein response: controlling cell fate decisions under ER stress and beyond. Nat. Rev. Mol Cell Biol. 13, 89–102 (2012). 22251901 10.1038/nrm3270

[b3] Xu C., Bailly-Maitre B. & Reed J. C. Endoplasmic reticulum stress: cell life and death decisions. J. Clin. Investig. 115, 2656–2664 (2005). 16200199 10.1172/JCI26373 PMC1236697

[b4] Rutkowski D. T. & Kaufman R. J. A trip to the ER: coping with stress. Trends Cell Biol. 14, 20–28 (2004). 14729177 10.1016/j.tcb.2003.11.001

[b5] Tabas I. & Ron D. Integrating the mechanisms of apoptosis induced by endoplasmic reticulum stress. Nat. Cell Biol. 13, 184–190 (2011). 21364565 10.1038/ncb0311-184 PMC3107571

[b6] Jing G., Wang J. J. & Zhang S. X. ER stress and apoptosis: a new mechanism for retinal cell death. Exp. Diab. Res. 2012, 589589 (2012). 10.1155/2012/589589 PMC3246718 22216020

[b7] Lin J. H. . IRE1 signaling affects cell fate during the unfolded protein response. Science 318, 944–949 (2007). 17991856 10.1126/science.1146361 PMC3670588

[b8] Chen Y. & Brandizzi F. IRE1: ER stress sensor and cell fate executor. Trends Cell Biol. 23, 547–555 (2013). 23880584 10.1016/j.tcb.2013.06.005 PMC3818365

[b9] Chen L. . Cab45S inhibits the ER stress-induced IRE1-JNK pathway and apoptosis via GRP78/BiP. Cell Death Dis. 5, e1219 (2014). 24810055 10.1038/cddis.2014.193 PMC4047922

[b10] Urano F. . Coupling of stress in the ER to activation of JNK protein kinases by transmembrane protein kinase IRE1. Science 287, 664–666 (2000). 10650002 10.1126/science.287.5453.664

[b11] Oslowski C. M. & Urano F. Measuring ER stress and the unfolded protein response using mammalian tissue culture system. Methods Enzymol. 490, 71–92 (2011). 21266244 10.1016/B978-0-12-385114-7.00004-0 PMC3701721

[b12] Lemaire K. . Ubiquitin fold modifier 1 (UFM1) and its target UFBP1 protect pancreatic beta cells from ER stress-induced apoptosis. PLoS ONE 6, e18517 (2011). 21494687 10.1371/journal.pone.0018517 PMC3071830

[b13] Hofmann K. & Bucher P. The PCI domain: a common theme in three multiprotein complexes. Trends Biochem. Sci. 23, 204–205 (1998). 9644972 10.1016/s0968-0004(98)01217-1

[b14] Wu J., Lei G., Mei M., Tang Y. & Li H. A novel C53/LZAP-interacting protein regulates stability of C53/LZAP and DDRGK domain-containing Protein 1 (DDRGK1) and modulates NF-kappaB signaling. J. Biol. Chem. 285, 15126–15136 (2010). 20228063 10.1074/jbc.M110.110619 PMC2865345

[b15] Tatsumi K. . A novel type of E3 ligase for the Ufm1 conjugation system. J Biol. Chem. 285, 5417–5427 (2010). 20018847 10.1074/jbc.M109.036814 PMC2820770

[b16] Kwon J. . A novel LZAP-binding protein, NLBP, inhibits cell invasion. J. Biol. Chem. 285, 12232–12240 (2010). 20164180 10.1074/jbc.M109.065920 PMC2852962

[b17] Azfer A., Niu J., Rogers L. M., Adamski F. M. & Kolattukudy P. E. Activation of endoplasmic reticulum stress response during the development of ischemic heart disease. Am. J. Physiol. Heart Circ. Physiol. 291, H1411–H1420 (2006). 16617122 10.1152/ajpheart.01378.2005 PMC1575464

[b18] Lu H., Yang Y., Allister E. M., Wijesekara N. & Wheeler M. B. The identification of potential factors associated with the development of type 2 diabetes: a quantitative proteomics approach. Mol. Cell. Proteom. 7, 1434–1451 (2008). 10.1074/mcp.M700478-MCP200 PMC2500228 18448419

[b19] Zhang Y., Zhang M., Wu J., Lei G. & Li H. Transcriptional regulation of the Ufm1 conjugation system in response to disturbance of the endoplasmic reticulum homeostasis and inhibition of vesicle trafficking. PLoS ONE 7, e48587 (2012). 23152784 10.1371/journal.pone.0048587 PMC3496721

[b20] Yoo H. M. . Modification of ASC1 by UFM1 is crucial for ERalpha transactivation and breast cancer development. Mol. Cell 56, 261–274 (2014). 25219498 10.1016/j.molcel.2014.08.007

[b21] Xi P., Ding D., Zhou J., Wang M. & Cong Y. S. DDRGK1 regulates NF-kappaB activity by modulating IkappaBalpha stability. PLoS ONE 8, e64231 (2013). 23675531 10.1371/journal.pone.0064231 PMC3651127

[b22] Janicke R. U. MCF-7 breast carcinoma cells do not express caspase-3. Breast Cancer Res. Treat. 117, 219–221 (2009). 18853248 10.1007/s10549-008-0217-9

[b23] Zhang K. . The unfolded protein response transducer IRE1alpha prevents ER stress-induced hepatic steatosis. EMBO J. 30, 1357–1375 (2011). 21407177 10.1038/emboj.2011.52 PMC3094110

[b24] Sun S. . IRE1alpha is an endogenous substrate of endoplasmic-reticulum-associated degradation. Nat. Cell Biol. 17, 1546–1555 (2015). 26551274 10.1038/ncb3266 PMC4670240

[b25] Komatsu M. . A novel protein-conjugating system for Ufm1, a ubiquitin-fold modifier. EMBO J. 23, 1977–1986 (2004). 15071506 10.1038/sj.emboj.7600205 PMC404325

[b26] van Galen P. . The unfolded protein response governs integrity of the haematopoietic stem-cell pool during stress. Nature 510, 268–272 (2014). 24776803 10.1038/nature13228

[b27] Hetz C., Martinon F., Rodriguez D. & Glimcher L. H. The unfolded protein response: integrating stress signals through the stress sensor IRE1alpha. Physiol. Rev. 91, 1219–1243 (2011). 22013210 10.1152/physrev.00001.2011

[b28] Walter P. & Ron D. The unfolded protein response: from stress pathway to homeostatic regulation. Science 334, 1081–1086 (2011). 22116877 10.1126/science.1209038

[b29] Merksamer P. I. & Papa F. R. The UPR and cell fate at a glance. J. Cell Sci. 123, 1003–1006 (2010). 20332117 10.1242/jcs.035832 PMC2844313

[b30] Zhang M. . RCAD/Ufl1, a Ufm1 E3 ligase, is essential for hematopoietic stem cell function and murine hematopoiesis. Cell Death Diff. 22, 1922–1934 (2015). 10.1038/cdd.2015.51 PMC4816109 25952549

[b31] Tatsumi K. . The Ufm1-activating enzyme Uba5 is indispensable for erythroid differentiation in mice. Nat. Commun. 2, 181 (2011). 21304510 10.1038/ncomms1182 PMC3105337

[b32] Cai Y. . UFBP1, a key component of the Ufm1 conjugation system, is essential for ufmylation-mediated regulation of erythroid development. PLoS Genet. 11, e1005643 (2015). 26544067 10.1371/journal.pgen.1005643 PMC4636156

[b33] Chen Z. . Wip1 deficiency impairs haematopoietic stem cell function via p53 and mTORC1 pathways. Nat. Commun. 6, 6808 (2015). 25879755 10.1038/ncomms7808

