# Supplementary material for: A critical role of DDRGK1 in endoplasmic reticulum homoeostasis via regulation of IRE1α stability
Source: Nat Commun. 2017 Jan 27;8:14186. doi: 10.1038/ncomms14186 (PMC5290148; doi:10.1038/ncomms14186)
Supplement: Supplementary Information — Supplementary figures and supplementary table. [file ncomms14186-s1.pdf]

Supplementary Information

Supplementary Figure 1

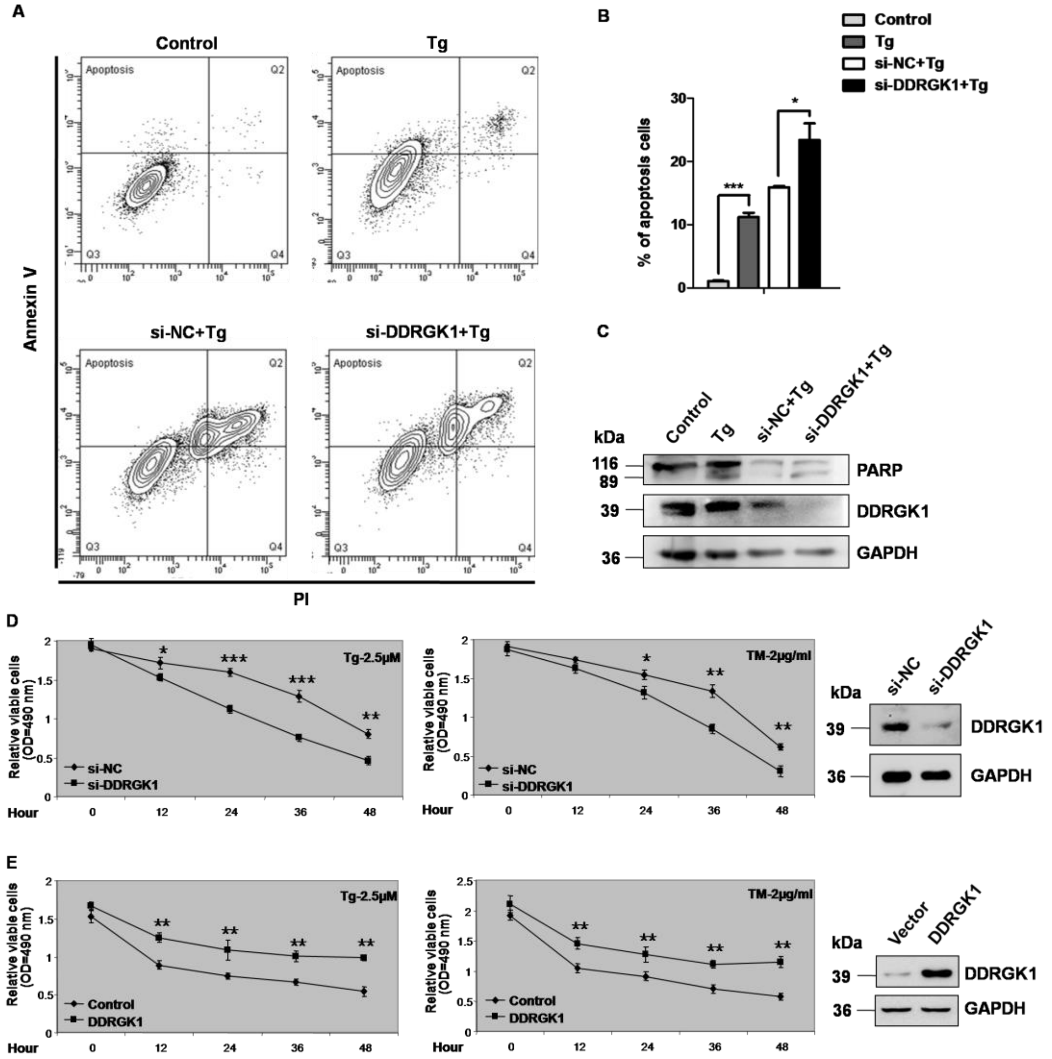

Supplementary Figure 1. DDRGK1 is an essential factor in ER homeostasis. A. MCF7 cells were transfected with siRNA control or siRNA against DDRGK1 for 72 h, cells were treated with DMSO (vehicle control) or Tg (2.5 μM) for 24 h as indicated before harvest. Cells were stained with Annexin V and PI followed by flow cytometric analysis. B. Quantification of apoptotic cells (Annexin V^+) in A. C. Western blot analysis of PARP in MCF7 cells described in A. D. MCF7 cells were transfected with siRNA control or with siRNA against DDRGK1 for 72 h, cells were treated with Tg (2.5 μM) or Tm (2 μg ml^{-1}) as indicated time (n=3), viable cells were measured by MTT assay. The knockdown efficiency of DDRGK1 in MCF7 cells was measured by western blot. E. MCF7 cells were transfected with control vector or DDRGK1 for 60 h, cells were treated with Tg (2.5 μM) or Tm (2 μg ml^{-1}) as indicated time (n=3), viable cells were measured by MTT assay.

The over-expression efficiency of DDRGK1 in MCF7 cells was measured by western blot. All data are presented as mean ± s.d. from three experiments. * p<0.05, ** p<0.01, and *** p<0.001 by Student's t-test.

Supplementary Figure 2

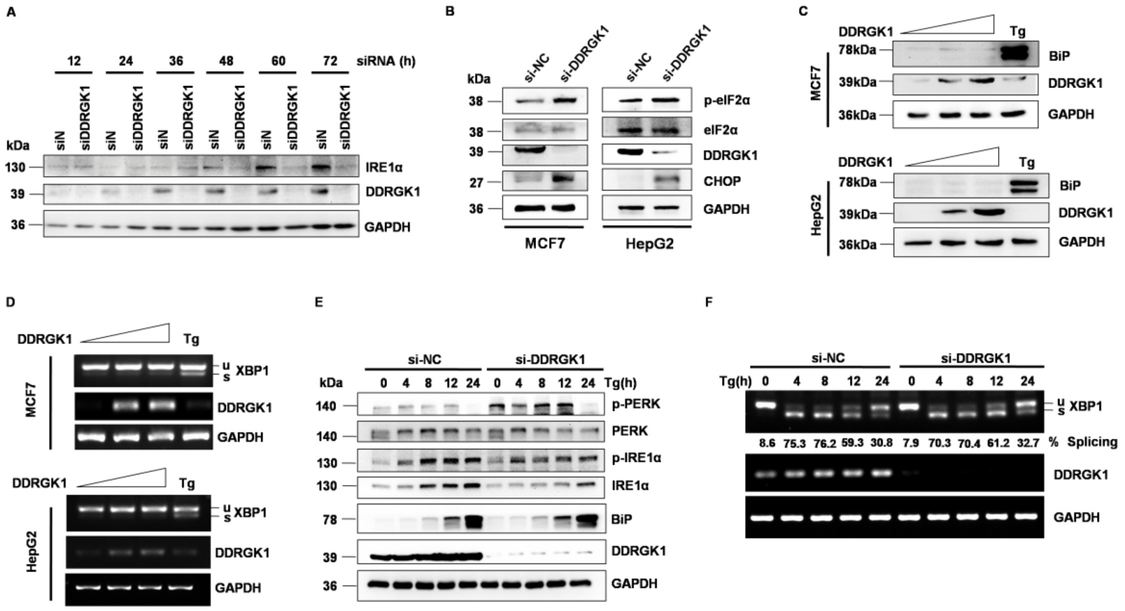

Supplementary Figure 2. DDRGK1 modulates the UPR. A. MCF7 cells were transfected with either control siRNA or siRNA targeting DDRGK1. The cells were harvested at the indicated time for western blot analysis of IRE1α protein levels. B. MCF7 and HepG2 cells were transfected with either control siRNA or siRNA targeting DDRGK1 for 72 h. The protein levels of p-eIF2α, eIF2α and CHOP were determined by western blot. C. MCF7 and HepG2 cells were transfected with control or DDRGK1 at various doses for 36 h. The protein level of BiP was determined by western blot (Tg treatment as a positive control). D. RT-PCR analysis of *XBP-1* splicing in C. E. HepG2 cells were transfected with siRNA control or siRNA targeting DDRGK1 for 72 h. Cells were collected for western blot after treatment with 2.5 μM Tg as the indicated time. F. RT-PCR analysis of *XBP-1* splicing in E.

Supplementary Figure 3

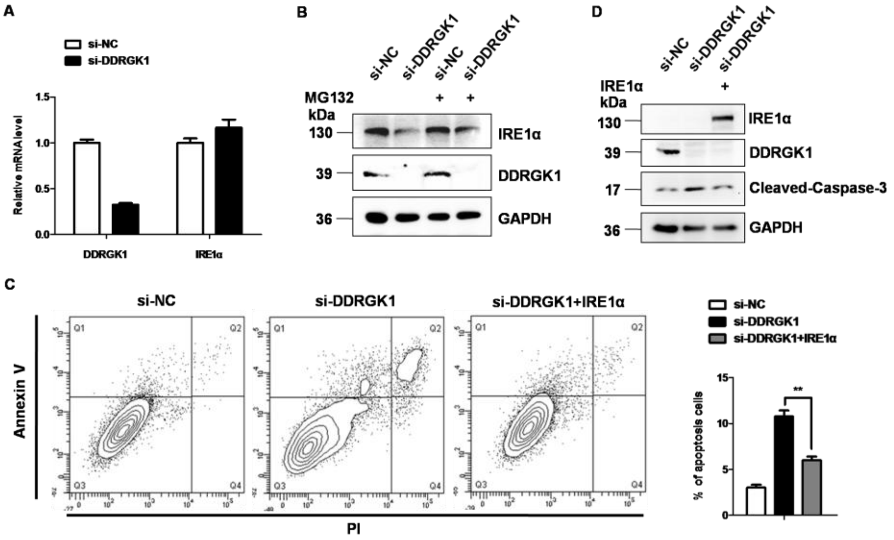

Supplementary Figure 3. DDRGK1 regulates UPR by targeting IRE1α. A. siRNA control and siRNA targeting DDRGK1 were transfected into MCF7 cells for 72 h. Q-PCR analysis of relative mRNA expression levels of *IRE1α*. B. Western blot analysis of IRE1α in control and DDRGK1-knockdown HepG2 cells treated with or without MG132 (20 μM, 8 h). C. HepG2 cells were transfected with either control siRNA or siRNA targeting DDRGK1 for 72 h. Before harvest, the DDRGK1-knockdown cells were transfected with either control or IRE1α for 36 h. The cells were subsequently stained with Annexin V and PI and subjected to flow cytometric analysis and followed by quantification of apoptotic cells (Annexin V^+). Data are presented as mean ± s.d. from three experiments. ** p<0.01 by Student's t-test. D. Western blot analysis of cleaved-Caspase-3 in HepG2 cells in figure C.

Supplementary Figure 4

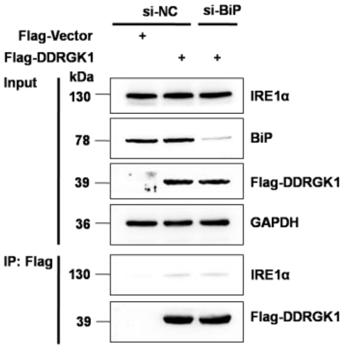

Supplementary Figure 4. DDRGK1 interacts with IRE1α independent of BiP. Western blot analysis of Flag M2 affinity gel immunoprecipitates in BiP-knockdown and control HEK293T cells transfected with Flag-Vector or Flag-DDRGK1 for 36 h.

Supplementary Figure 5

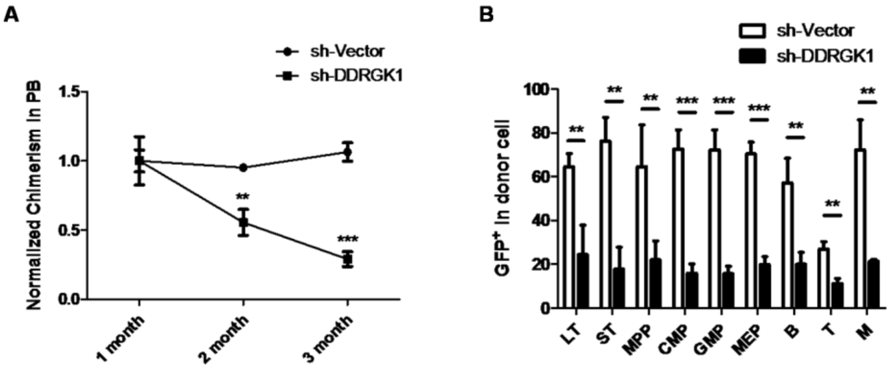

Supplementary Figure 5. Knockdown of DDRGK1 impairs the reconstitution ability of murine HSCs. A. The values represent the normalized percentages of donor-derived GFP^+ cells in the total engraftment (n=3). B. The values represent the percentages of donor-derived GFP^+ cells in LT-HSC, ST-HSC, MPP, CMP, GMP, MEP, B, T and myeloid lineage cell populations in the BM from primary recipient mice at 12 weeks after transplantation (n=3). All data are presented as mean ± s.d. ** p<0.01, and *** p<0.001 by Student's t-test.

Supplementary Figure 6

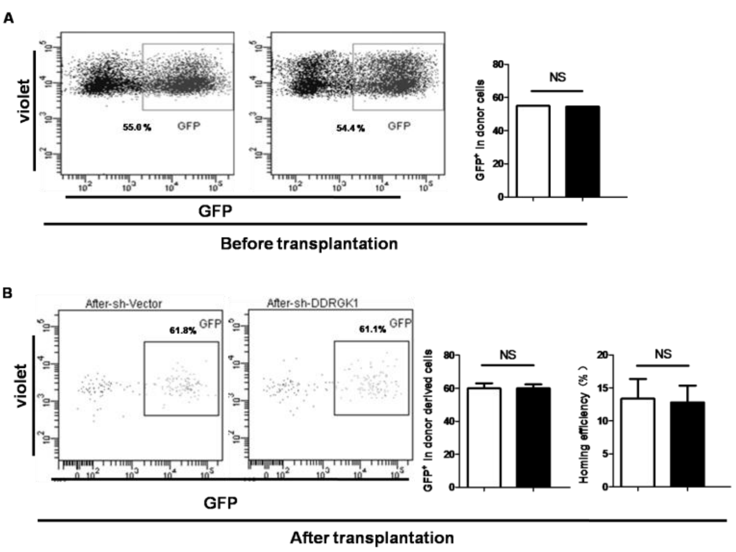

Supplementary Figure 6. Knockdown of DDRGK1 has no effect on homing ability of murine HSCs. A-B. Equal numbers of Violet$^+$ sh-Vector or sh-DDRGK1 lentiviral transduced LSK cells were transplanted into lethally irradiated WT recipients, and BM content was assessed 18 hours post-transplant. Representative flow cytometric analysis and bar graphs of the percentage of GFP$^+$ cells in Violet$^+$ control and DDRGK1-knockdown cells before and after transplantation were shown. Homing efficiency=absolute number of dye$^+$GFP$^+$ cells found in the BM 18 hours post-transplant ÷ absolute number of dye$^+$GFP$^+$ cells transplanted (n=5). All data are presented as mean ± s.d. NS, non-significant, by Student's *t*-test.

Supplementary Figure 7

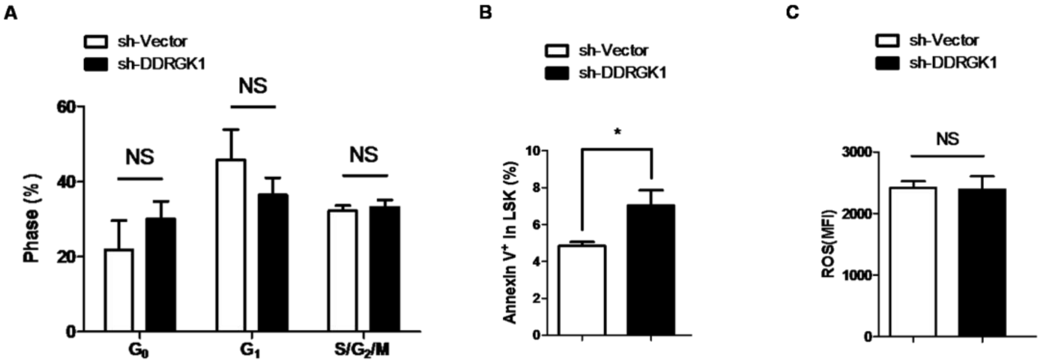

Supplementary Figure 7. Knockdown of DDRGK1 induces apoptosis in murine HSCs. A. Cell-cycle analysis GFP$^+$ LSK cells from control and DDRGK1-knockdown recipient mice was performed by staining with DAPI and Ki67 and analyzed by FACS (n=4). B. Bar graph shows the percentage of Annexin V positive cells in donor-derived control and DDRGK1-knockdown LSK cells after transplantation (n=3). C. Average MFI values in the LSK population in donor-derived control and DDRGK1-knockdown LSK cells after transplantation (n=3). All data are presented as mean ± s.d. * p<0.05; NS, non-significant, by Student's *t*-test.

Supplementary Figure 8

Uncropped gel images

Figure 1

Figure 1B

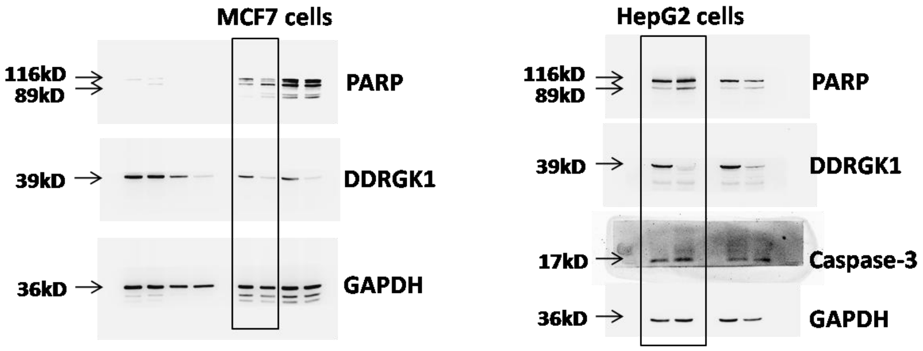

Figure 2

Figure 2C

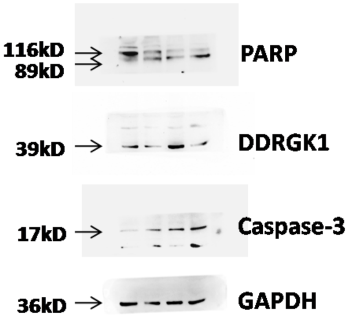

Figure 2F

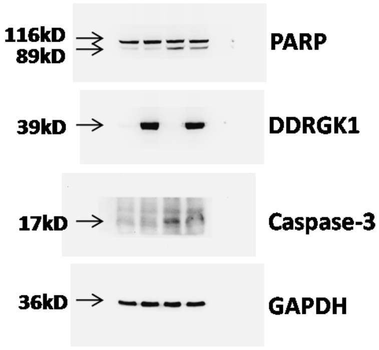

Figure 3

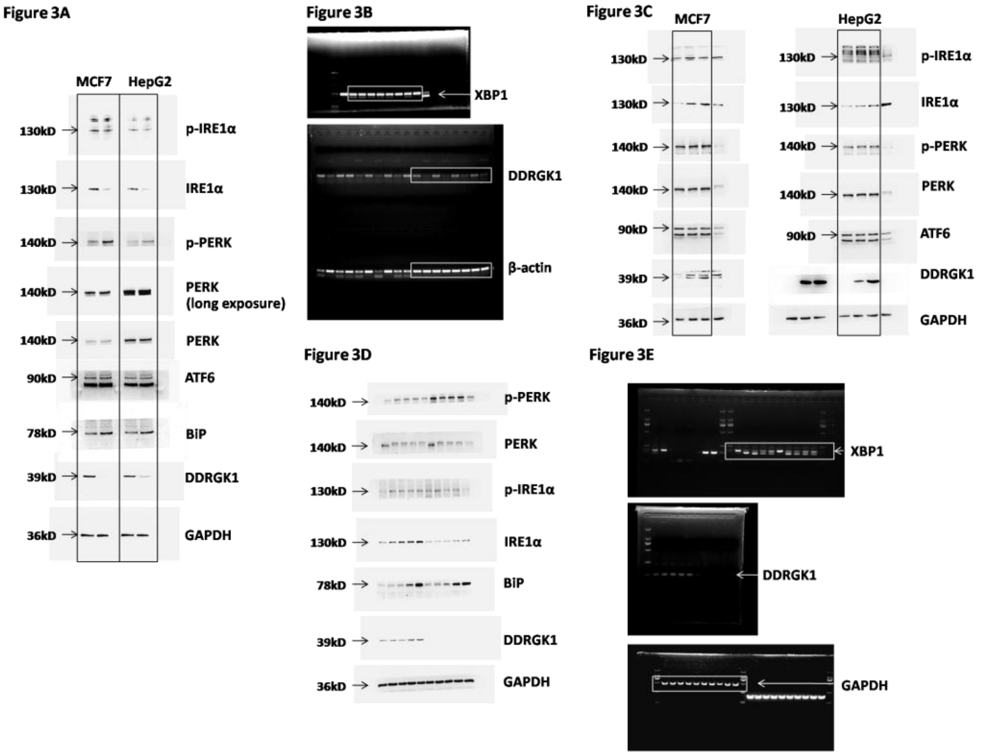

Figure 4

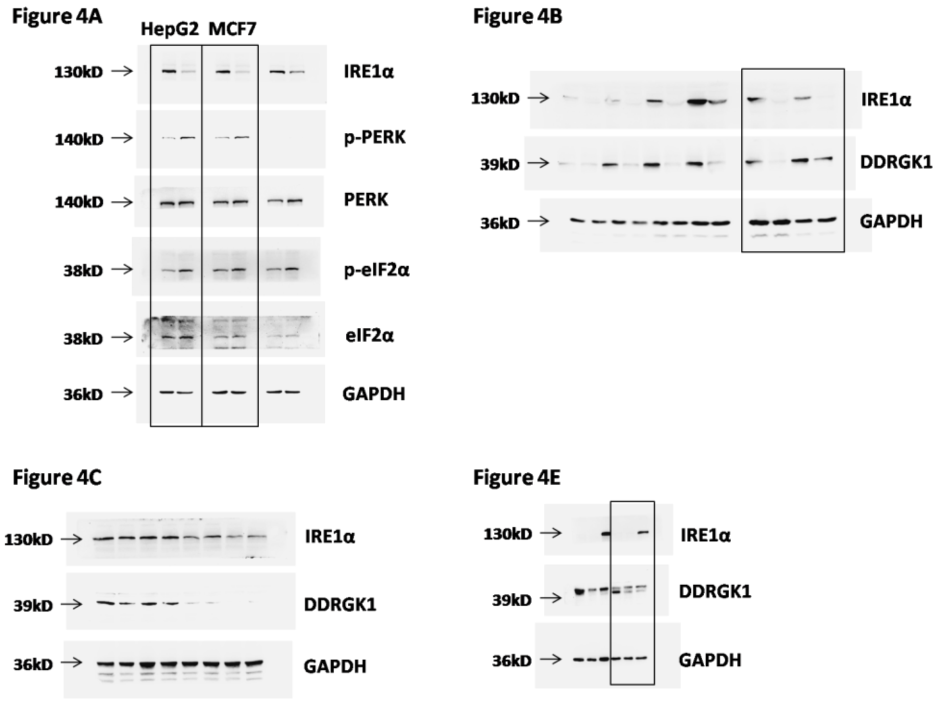

Figure 5

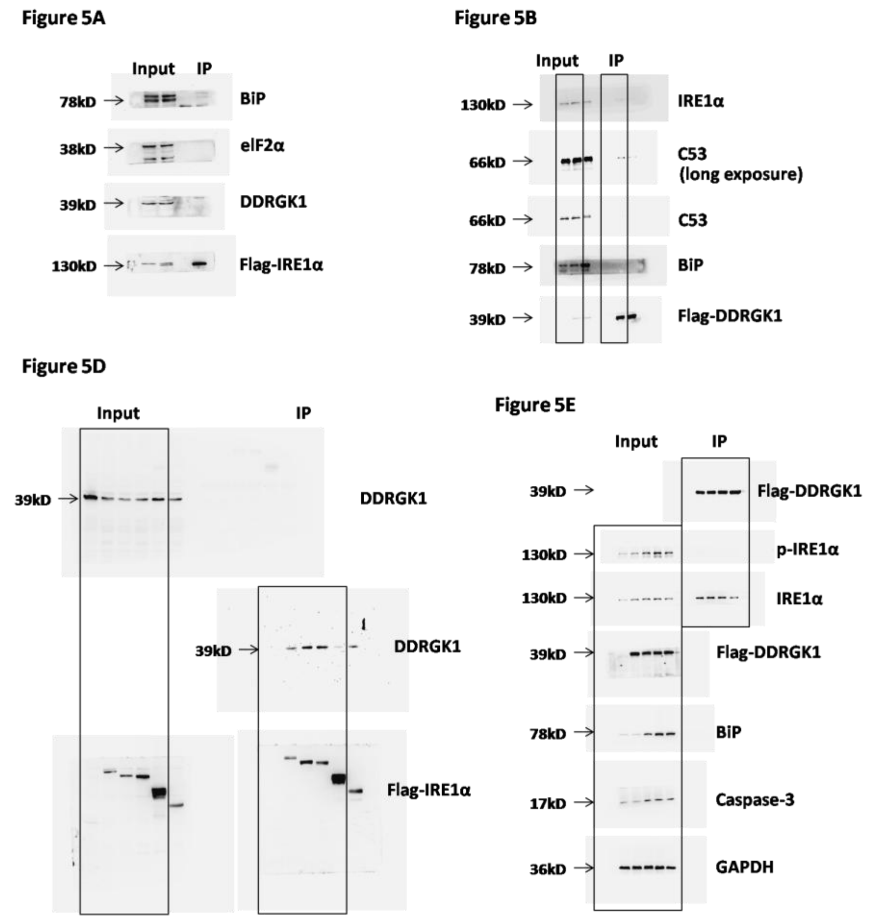
Figure 5A
Input
IP
78kD
BiP
38kD
eIF2α
39kD
DDRGK1
130kD
Flag-IRE1α
Figure 5B
Input
IP
130kD
IRE1α
66kD
C53
(long exposure)
66kD
C53
78kD
BiP
39kD
Flag-DDRGK1
Figure 5D
Input
IP
39kD
DDRGK1
39kD
DDRGK1
Flag-IRE1α
Figure 5E
Input
IP
39kD
Flag-DDRGK1
130kD
p-IRE1α
130kD
IRE1α
39kD
Flag-DDRGK1
78kD
BiP
17kD
Caspase-3
36kD
GAPDH

Figure 6

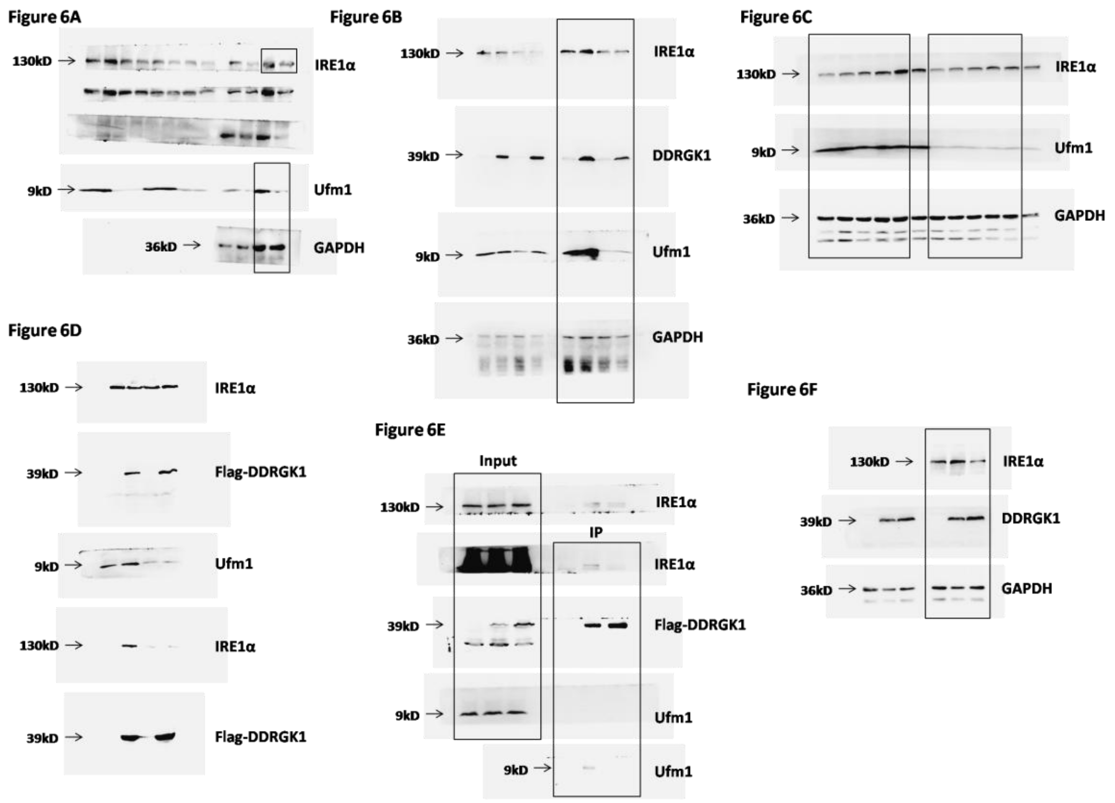

Figure 7

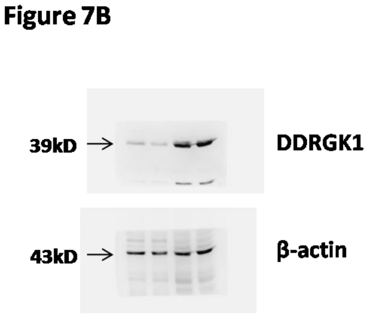

Figure 8

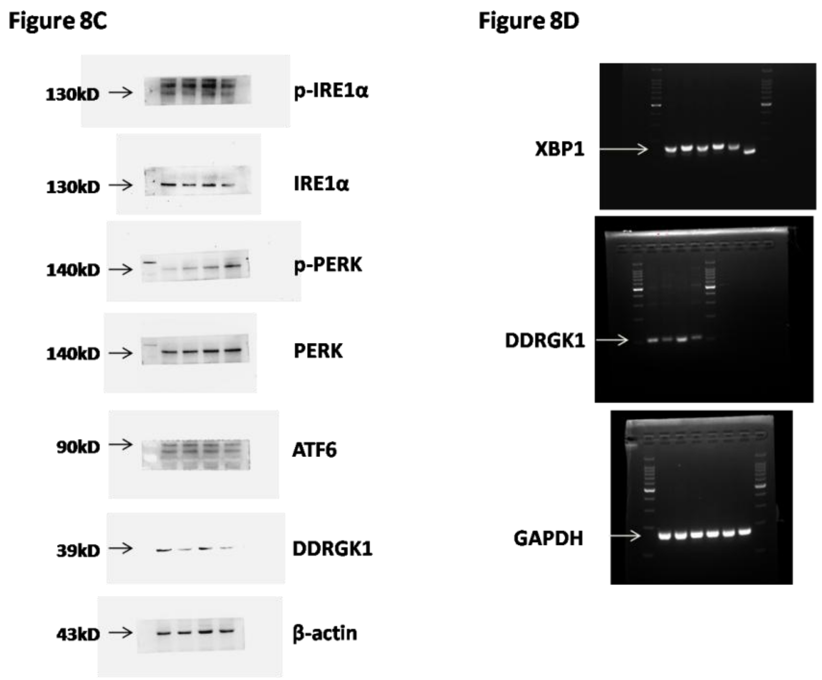

Supplementary Figure 1

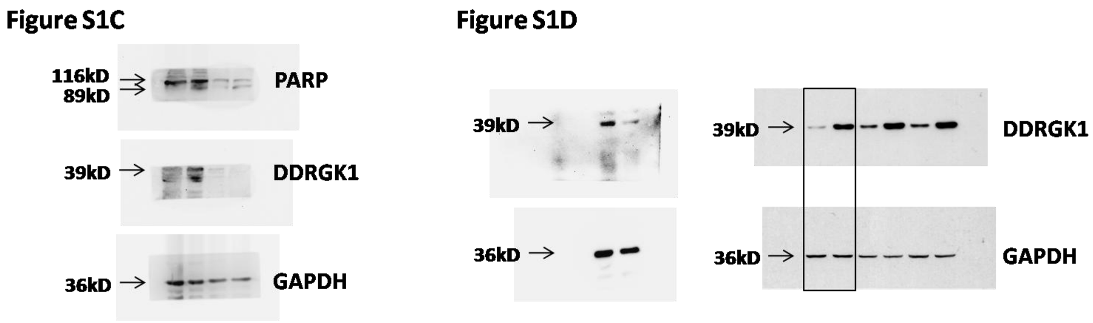

Supplementary Figure 2

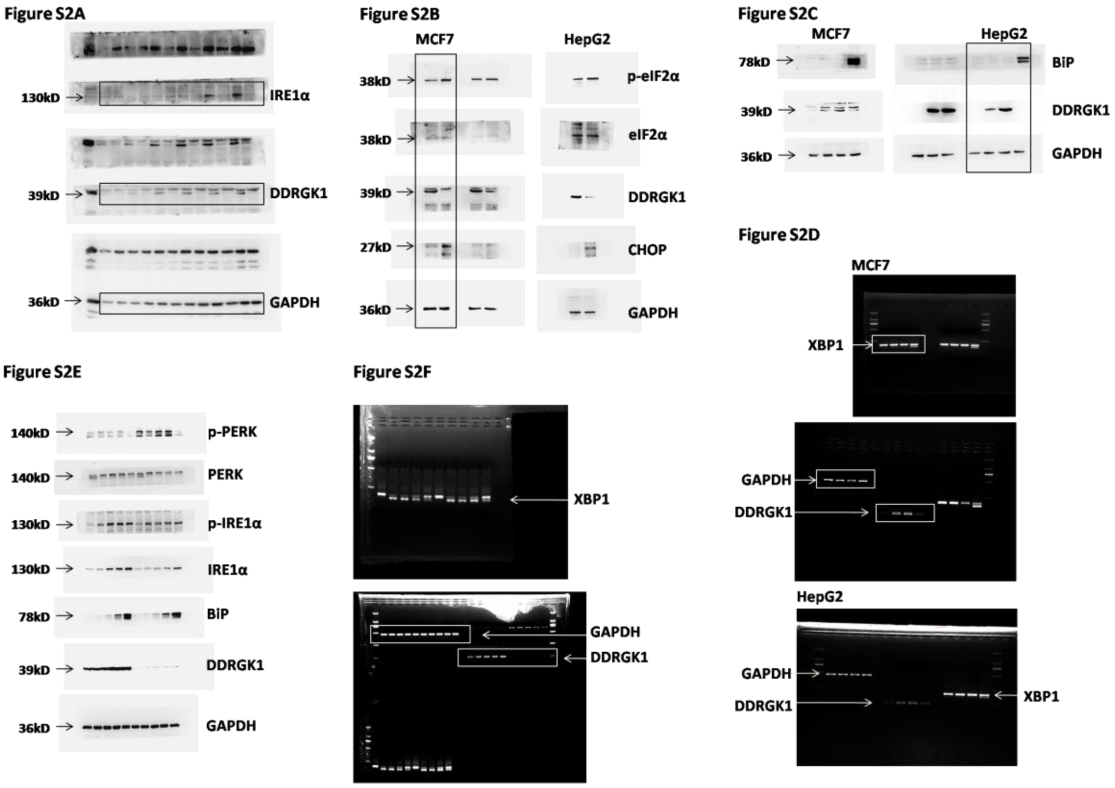

Supplementary Figure 3

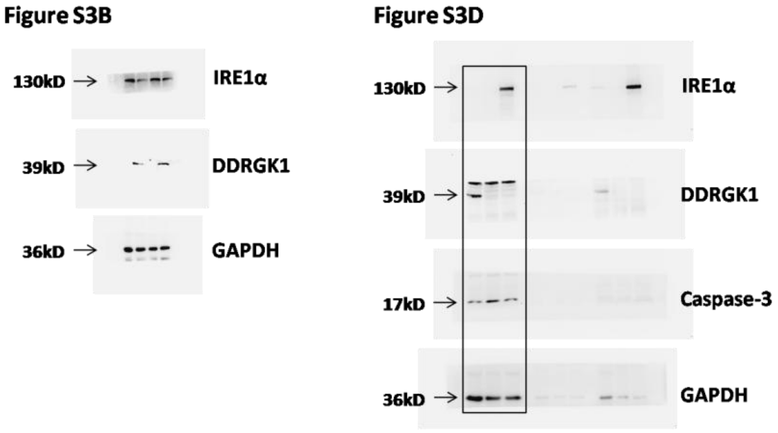

Supplementary Figure 4

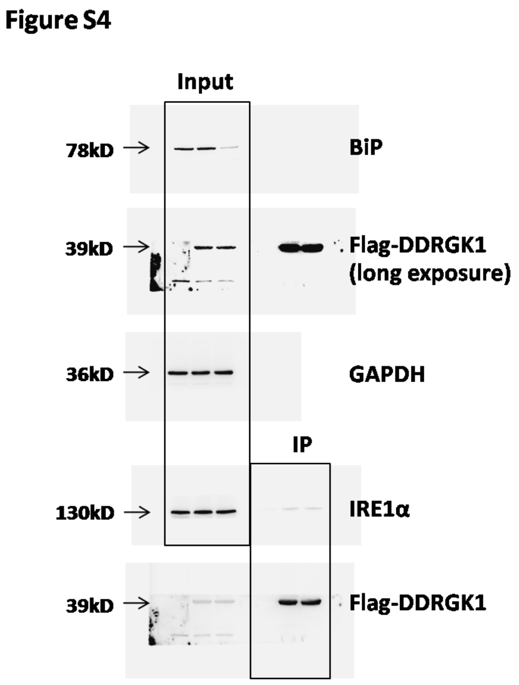

Supplementary Table 1.

The sequences of the primers employed for Q-PCR/RT-PCR as follows

Name (species)	Primers Sequences (5’→3’)
DDRGK1(h)	F-GGTTGTGCTCTTGGAAGACCT; R-TCACACCTGTTATAGTCCCCTC
BAX(h)	F-CCCGAGAGGTCTTTTTCCGAG; R-CCAGCCCATGATGGTTCTGAT
BAK(h)	F-ATGGTCACCTTACCTCTGCAA; R-TCATAGCGTCGGTTGATGTCG
NOXA(h)	F-ACCAAGCCGGATTTGCGATT; R-ACTTGCACTTGTTCCTCGTGG
Bid(h)	F-ATGGACCGTAGCATCCCTCC; R-GTAGGTGCGTAGGTTCTGGT
DR-5(h)	F-GCCCCACAACAAAAGAGGTC; R-AGGTCATTCCAGTGAGTGCTA
Bcl-2(h)	F-GGTGGGGTCATGTGTGTGG; R-CGGTTCAGGTACTCAGTCATCC
IRE1α(h)	F-CATCCCCATGCCGAAGTTCA; R-CTGCTTCTCTCCGGTCAGGA
HSPA8(h)	F-ACCTACTCTTGTGTGGGTGTT; R-GACATAGCTTGGAGTGGTTCG
BiP(h)	F-CATCACGCCGTCCTATGTCG; R-CGTCAAAGACCGTGTTCTCG
XBP-1(h)	F-CCGGTCTGCTGAGTCCGCAGC; R-TGGCAGGCTCTGGGGAAGGG
CHOP(h)	F-AAGGAAAGTGGCACAGCTAGCT; R-CTGGTCAGGCGCTCGATTT

GAPDH(h)	F-GGAGCGAGATCCCTCCAAAAT; R-GGCTGTTGTCATACTTCTCATGG
β -actin(h)	F-AGAGCTACGAGCTGCCTGAC;<br>R-AGCACTGTGTTGGCGTACAG
18S(h)	F-GGACACGGACAGGATTGACA; R-GACATCTAAGGGCATCACAG
DDRGK1(m)	F-GAGCACGAGGAGTACCTGAAA; R-TCCTGAGTCCTTAGGCCCATC
BiP(m)	F-GAAAGGATGGTTAATGATGCTGAGAAG;<br>R-GTCTTCAATGTCCGCATCCTG
CHOP(m)	F-CATACACCACCACACCTGAAAG; R-CCGTTTCCTAGTTCTTCCTTGC
XBP-1(m)	F-ACACGCTTG GGAAT GACAC; R- CCATGGGAAGATGTTCTGGG
β -actin(m)	F-CTCAGGAGGAGCAATGATCTTGAT; R-TACCACCATGTACCCAGGCA
GAPDH(m)	F-CGACTTCAACAGCAACTCCCACTCTTCC;<br>R-TGGGTGGTCCAGGGTTTCTTACTCCTT
